# Plastid dsRNA transgenes trigger phased small RNA-based gene silencing of nuclear-encoded genes

**DOI:** 10.1093/plcell/koad165

**Published:** 2023-06-13

**Authors:** Sébastien Bélanger, Marianne C Kramer, Hayden A Payne, Alice Y Hui, R Keith Slotkin, Blake C Meyers, Jeffrey M Staub

**Affiliations:** Donald Danforth Plant Science Center, 975 N. Warson Road, St. Louis, MO 63132, USA; Donald Danforth Plant Science Center, 975 N. Warson Road, St. Louis, MO 63132, USA; Donald Danforth Plant Science Center, 975 N. Warson Road, St. Louis, MO 63132, USA; Plastomics Inc, 1100 Corporate Square Drive, St. Louis, MO 63132, USA; Donald Danforth Plant Science Center, 975 N. Warson Road, St. Louis, MO 63132, USA; Division of Biological Sciences, University of Missouri, Columbia, MO 65211, USA; Donald Danforth Plant Science Center, 975 N. Warson Road, St. Louis, MO 63132, USA; Division of Plant Science and Technology, University of Missouri, Columbia, MO 65211, USA; Plastomics Inc, 1100 Corporate Square Drive, St. Louis, MO 63132, USA

## Abstract

Plastid transformation technology has been widely used to express traits of potential commercial importance, though the technology has been limited to traits that function while sequestered in the organelle. Prior research indicates that plastid contents can escape from the organelle, suggesting a possible mechanism for engineering plastid transgenes to function in other cellular locations. To test this hypothesis, we created tobacco (*Nicotiana tabacum* cv. Petit Havana) plastid transformants that express a fragment of the nuclear-encoded *Phytoene desaturase* (*PDS*) gene capable of catalyzing post-transcriptional gene silencing if RNA escapes into the cytoplasm. We found multiple lines of direct evidence that plastid-encoded *PDS* transgenes affect nuclear *PDS* gene silencing: knockdown of the nuclear-encoded *PDS* mRNA and/or its apparent translational inhibition, biogenesis of 21-nucleotide (nt) phased small interfering RNAs (phasiRNAs), and pigment-deficient plants. Furthermore, plastid-expressed dsRNA with no cognate nuclear-encoded pairing partner also produced abundant 21-nt phasiRNAs in the cytoplasm, demonstrating that a nuclear-encoded template is not required for siRNA biogenesis. Our results indicate that RNA escape from plastids to the cytoplasm occurs generally, with functional consequences that include entry into the gene silencing pathway. Furthermore, we uncover a method to produce plastid-encoded traits with functions outside of the organelle and open additional fields of study in plastid development, compartmentalization, and small RNA biogenesis.

## Teaser

Plastid-expressed double-strand RNA can escape the chloroplast compartment, triggering 21-nt phasiRNA biogenesis in the cytoplasm, resulting in silencing of targeted nuclear genes.

**IN A NUTSHELL**

**Background:** Plants have 3 genomes: the nuclear, the mitochondrial, and the chloroplast genomes. The chloroplast is maternally inherited, so transgene insertion into the chloroplast genome prevents its dissemination into the environment. We know that dsRNA expressed from the chloroplast can be used for cross-kingdom RNA interference (RNAi)–based repression of pathogenic insects by knockdown of essential genes in those pests.

**Question:** Can dsRNAi transgenes expressed in chloroplasts regulate the expression of nuclear-encoded genes in the host plant via an RNAi pathway? We hypothesized that dsRNA molecules may escape from the organelle during development and enter the cytoplasmic gene silencing pathway to repress nuclear-encoded mRNAs.

**Findings:** We inserted a dsRNA transgene into the chloroplast genome that carries a fragment of the nuclear gene encoding phytoene desaturase (PDS), which is essential for carotenoid and chlorophyll accumulation. Plants carrying this transgene in their chloroplasts showed a white “bleached” phenotype indicative of little or no carotenoid and chlorophyll accumulation at different stages of plant development. This indicates that the dsRNA transgene expressed by the chloroplast can silence the expression of the nuclear gene. Unexpectedly, we found that plastid-expressed dsRNA transgenes produced 21-nucleotide phased short interfering small RNAs (phasiRNAs) in the cytoplasm. Interestingly, the plastid-expressed dsRNA caused gene silencing via a small RNA biogenesis pathway that includes “untriggered siRNAs.” Our results provide insight into the biosynthesis of phasiRNAs and RNA biology and open potential opportunities for chloroplast engineering to modulate the expression of nuclear genes and thus affect plant traits.

**Next steps:** We want to understand how dsRNA transgenes expressed from the chloroplast genome enter the cytoplasm and bypass the initial biosynthetic steps of the phasiRNA pathway.

## Introduction

Plastid transformation technology was first developed in the model crop tobacco (*Nicotiana tabacum*) nearly 30 years ago (Svab et al. 1990; Svab and Maliga 1993). Plastid transformation is an attractive technology for potential commercialization of crops engineered with biotechnology traits for several reasons: introduction of traits into the plastid genome via homologous recombination facilitates trait gene stacking, the possibility for high level transgene expression, especially in the abundant chloroplasts of leaves, and natural transgene containment due to maternal inheritance of plastids in most crop plants (Maliga 2004; Bock 2015; Greiner et al. 2015; Maliga and Bock 2011). The technology has been used for expression of transgenes that impart useful traits such as insect control, herbicide tolerance, introduction of high value molecules, and metabolic pathways to enhance nutritional value (Ye et al. 2001; Dufourmantel et al. 2007; Zhang et al. 2017; Bally et al. 2018; Apel and Bock 2009; Staub et al. 2000). Plastid transformation has been reported in numerous plant species, including commercial row crops like soybean (*Glycine max*) (Dufourmantel et al. 2004), although is currently routine only in multiple Solanaceae.

dsRNA expressed in plastids has been shown to efficiently catalyze post-transcriptional gene silencing [PTGS or RNA interference (RNAi)] in insect pests (Zhang et al. 2015; Bally et al. 2016; Jin et al. 2015; Dong et al. 2022; Wu et al. 2022, 2023). High-level accumulation and efficacy of long insecticidal dsRNA in plastids were attributed to the lack of dsRNA processing in plastids due to the absence of the RNAi machinery (Bally et al. 2016; Jin et al. 2015), while processing of the dsRNA to insecticidal small RNAs occurs in insect gut cells after ingestion of leaf material. The gene silencing components are encoded in the plant by nuclear genes with their activities taking place both in the nucleus and cytoplasm (Kumakura et al. 2009; Montavon et al. 2018). Typically, plant nuclear-encoded dsRNA transgenes may lose efficacy due to processing of their dsRNA in the cytoplasm to 21 to 24 nucleotide (nt) small interfering RNAs (siRNAs), which are not efficiently taken up by insect gut cells (Li et al. 2015; Bolognesi et al. 2012). siRNA processing occurs on membrane-bound polysomes to trigger the production of secondary siRNAs that are generated on the rough endoplasmic reticulum (Li et al. 2016). These secondary siRNAs have a characteristic “phased” pattern, resulting from successive cleavage by a DICER-LIKE protein on the dsRNA substrate. In many plants, DICER-LIKE4 generates 21-nt phased siRNAs (phasiRNAs), which are a characteristic outcome of PTGS (Liu et al. 2020).

An assumed limitation of plastid engineering is that the transgene function is sequestered in the organelle. Thus, metabolic pathways in the cytoplasm or regulatory functions in the nucleus have not been amenable to plastid engineering. Although plastids import up to ∼3,000 nuclear-encoded proteins (Sjuts et al. 2017; Paila et al. 2015), there is no known mechanism for plastid-encoded gene products to leave or function outside of the organelle. Despite this, there is evidence to suggest that plastid-encoded proteins and nucleic acids can be found in other cellular compartments. For example, during the natural process of chloroplast autophagy, Rubisco and other chloroplast-encoded proteins transit to or in vacuoles prior to protein degradation (Otegui 2017; Izumi et al. 2015).

Intact plastid-encoded RNAs have not been observed outside of the organelle, though plastid-encoded tRNA fragments have been observed in the cytoplasm (Cognat et al. 2017; Alves and Nogueira 2021). Over evolutionary time frames, plastid genes have migrated to the nucleus (McFadden 2001) and can “escape” during plastid or mitochondrial transformation experiments when strong selection is used to identify rare transfer of DNA from the organelle to the nucleus (Fuentes et al. 2012; Stegemann and Bock 2006; Stegemann et al. 2003; Thorsness and Fox 1990; Wang et al. 2018). These observations suggest a means by which plastid DNA or RNA can exit the organelle to function in other cellular compartments.

In this study, we tested whether plastid-expressed transgenic RNAs can escape the organelle and function in the cytoplasmic PTGS pathway. We created tobacco plastid transformants that express a fragment of the nuclear-encoded *Phytoene desaturase* (*PDS*) gene, for which knockdown or translational inhibition of its cytoplasmic-localized mRNA results in an easily discernible, pigment deficient phenotype (Senthil-Kumar et al. 2007; Busch et al. 2002). We expressed dsRNA, sense, and antisense transcripts against *PDS* in transplastomic tobacco and found that the plastid-encoded transgenes affect nuclear *PDS* gene silencing. Knockdown of nuclear-encoded *PDS* was detected by reverse transcription quantitative PCR (RT-qPCR), and small RNA sequencing indicated processing of the plastid-expressed dsRNA into 21-nt phasiRNAs, giving direct evidence for a PTGS mechanism. Interestingly, transplastomic lines that express dsRNA against insect gene targets, for which no pairing partner is encoded in the nuclear genome of tobacco, are also processed to phasiRNAs in the cytoplasm. Our results indicate a common process of RNA escape from plastids to the cytoplasm that can be exploited for knockdown of host nuclear-encoded genes and expands the repertoire of biotechnology tools afforded by plastid transformation technology.

## Results

### Integration of a PDS cDNA fragment in the tobacco plastid genome

We first tested the capability of a plastid-encoded fragment of the *PDS* gene to silence its nuclear-encoded, cytoplasm-localized transcript. Two genomic loci (Nitab4.5_0006338g0050.1 and Nitab4.5_0004950g0020.1; Supplemental Fig. S1A), designated here as *PDS1* and *PDS2*, respectively, encoding *PDS* coding regions with >99% nt sequence identity were identified in tobacco (*N. tabacum*). We selected a 294-nt region of the *PDS1* cDNA with 7-nt polymorphisms compared to *PDS2* to enable subsequent discrimination of the multiple *PDS* genes. Further, we introduced a small 13-nt insertion sequence into the plastid *PDS* transgenes to discriminate them from the otherwise identical nuclear-encoded *PDS* genes (Supplemental Fig. S1B).

**Figure 1.**
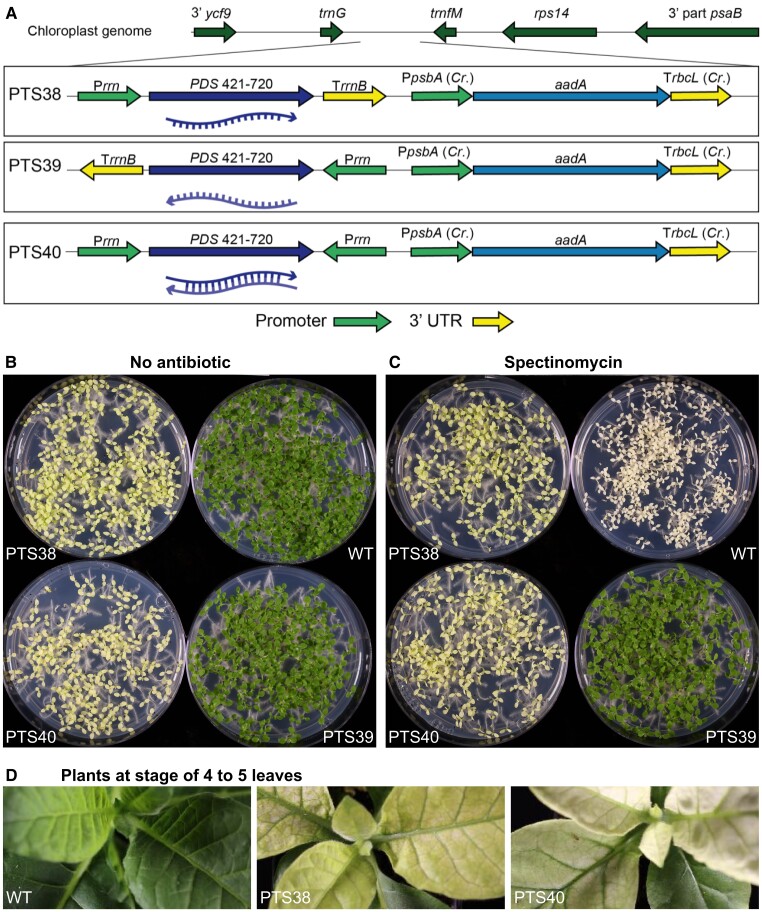
Plastid-expressed sense and double-strand RNA PDS transgenes produce pigment deficient tobacco seedlings. **A)***PDS* sense RNA (PTS38), antisense RNA (PTS39), and dsRNA (PTS40) transgenes and the *aadA* selectable marker are integrated into the plastid genome between the resident *trnG* and *trnfM* plastid genes via homologous flanking regions. Oligonucleotide probes used for *PDS* gene detection are shown by the red lines. **B, C)** Twelve-day-old T1 seedlings derived from self-fertilized T0 plants are sown on media lacking antibiotics **B**) or containing spectinomycin **C**). **D)** T1 green plants grown for ∼6 weeks in tissue culture and then placed in soil show rapid bleaching of new leaves. UTR, untranslated region; P*rrn*, promoter of the plastid 16SrDNA gene; T*rrnB*, *Escherichia coli rrnB* terminator fragment; P*psbA*, promoter of the plastid *psbA* gene; T*rbcL*, 3′end derived from the plastid *rbcL* gene; *aadA,* spectinomycin resistance gene; *Cr.*, *Chlamydomonas reinhardtii.* WT, wild-type; PTS, *plastid transformed lines carrying tobacco PDS transgenes*.

To determine if sense, antisense, or dsRNA transcripts could cause silencing of the nuclear-encoded gene, we designed 3 plastid transgenes. The PTS40 plastid transformation vector expresses the *PDS1* cDNA fragment from 2 convergent plastid (P*rrn*) promoters (Fig. 1A) that were previously shown to drive high-level accumulation of unprocessed dsRNA in chloroplasts (Zhang et al. 2015). As controls, both sense and antisense orientations of the *PDS1* cDNA fragment were expressed from a single plastid P*rrn* promoter and used the *Escherichia coli rrnB* 3′-end (T*rrn*; Zhang et al. 2015), to create PTS38 and PTS39, respectively (Fig. 1A). The *PDS1* transgenes were cloned next to a selectable *aadA* spectinomycin resistance gene and between regions with identity to the tobacco plastid genome to mediate site-directed integration by homologous recombination (Fig. 1A).

Tobacco plastid transformation of in vitro-grown leaf tissue was used to introduce the transgenes via homologous recombination into the plastid genome. Tissue from transformed shoots was used for 2 subsequent rounds of plant regeneration to ensure homoplasmy of the plastid transformed lines. Integration of the transgenes was confirmed in all transformed lines during the selection process by sequencing of PCR amplicons. Site-directed integration and homoplasmy of the transgenes were confirmed by DNA gel blot analysis (Supplemental Fig. S1C).

### Transplastomic plants exhibit pigment-deficient phenotypes

T0 plastid-transformed (transplastomic) lines grew normally in sterile tissue culture and had no apparent off-types. Homoplasmic plants were rooted in tissue culture, transferred to soil, and placed in a growth chamber with cycling light to acclimate plants for subsequent greenhouse growth. After ∼10 days in soil, new leaves from PTS38 (sense) and PTS40 (dsRNA) lines, but not PTS39 (antisense) lines, were observed to be pigment deficient. The pigment-deficient phenotype appeared uniform in new leaves, though with a yellow or very pale green appearance, suggesting the presence of residual chlorophyll and/or other carotenoids. As the transplastomic plants continued to grow, subsequent new leaves were either uniformly bleached or appeared chimeric with irregular patterns of bleaching mixed with some nearly green sectors (Fig. 1D). In contrast, the PTS39 *PDS* antisense line was uniformly green and had no apparent phenotype, as in wild-type controls. All independent transplastomic events for each of the constructs that were transferred to soil had similar phenotypes, indicating the observed pigment deficiency was caused by the plastid transgenes. These phenotypes were consistent across lines and generations, as described below.

The pigment deficiency observed in the transplastomic plants suggested a knockdown of the nuclear-encoded *PDS* gene function. Knockdown of nuclear-encoded *PDS* genes via nuclear transformation technologies typically results in albino leaf tissues (for example, Senthil-Kumar et al. 2007) due to lack of carotenoid accumulation and concomitant degradation of chlorophyll, suggesting some differences between plastid-expressed *PDS* gene silencing and the previously observed nuclear gene silencing approaches. Although the pigment-deficient lines grew much more slowly than green plants, they eventually out-grew the bleaching phenotype and were transferred to the greenhouse, where lateral branching occurred and flowering was ultimately profuse with an apparently normal seed set.

### Inheritance of plastid-encoded traits

To confirm maternal inheritance of the plastid-encoded traits, antibiotic resistance and pigment deficient phenotypes of seedlings from self-fertilized transplastomic lines were analyzed. As expected, wild-type seedlings were green on medium lacking antibiotics (Fig. 1B) but were uniformly bleached white on medium containing spectinomycin (Fig. 1C), indicating their sensitivity to the antibiotic. In contrast, seedlings derived from self-fertilization of the PTS38 and PTS40 lines had an intermediate phenotype, uniformly bleached yellow rather than white when grown on plates with or without spectinomycin (Fig. 1, B and C). These results indicate that the PTS38 and PTS40 lines are resistant to the antibiotic and homoplasmic and that the pigment deficiency may be due to silencing of the nuclear-encoded *PDS* genes. Seedlings of the PTS39 antisense line were uniformly green, indicating uniform resistance to the antibiotic, as expected for a homoplasmic, plastid-encoded trait, but no pigment deficiency was observed in these lines (Fig. 1, B and C).

**Figure 2.**
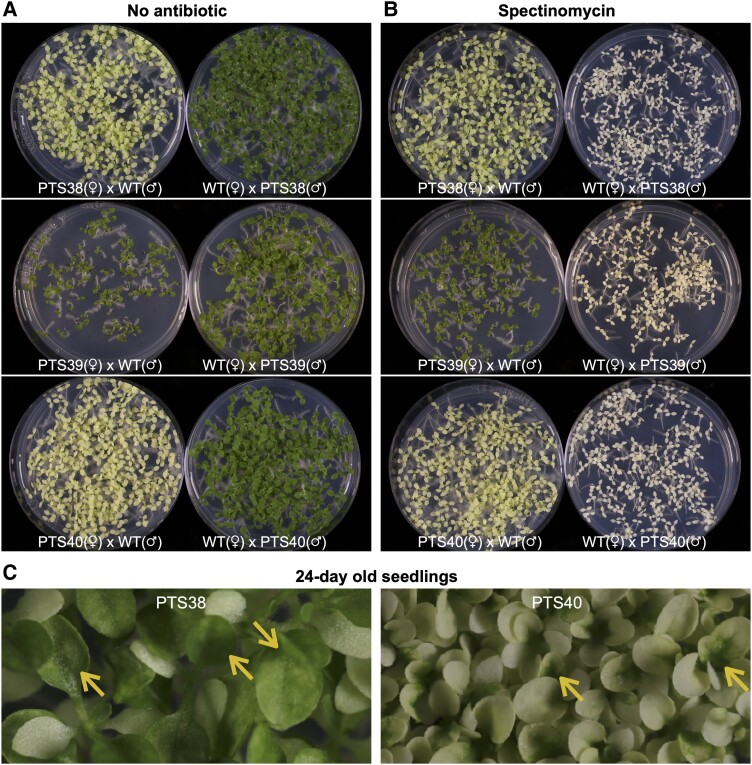
Maternal inheritance of plastid-encoded pigment deficiency. **A, B)** T1 seedlings derived from reciprocal crosses of T0 transplastomic plants to wild-type plants were sown in the absence **A**) or presence **B**) of spectinomycin antibiotic. **C)** While cotyledons are completely bleached in the PTS38 and PTS40 seedlings, the first true leaves become green (yellow arrows). WT, wild-type; PTS, plastid transformed lines carrying tobacco *PDS* transgenes; T0 and T1, transgenic generations; ♂, male; ♀, female.

Reciprocal crosses of plastid-transformed lines with wild-type plants are used to test maternal inheritance of a plastid transgenic trait (Svab et al. 1990; Staub and Maliga 1992). When the PTS38 and PTS40 plants were used as female parents in crosses to wild-type plants, all seedlings were uniformly pigment deficient on media lacking antibiotics (Fig. 2A). In contrast, when the plastid-transformed lines were used as the male pollen donor in crosses to wild-type plants, all seedlings were uniformly green (Fig. 2A). In the presence of the selective antibiotic (Fig. 2B), all female-derived transplastomic seedlings were also pigment deficient but uniformly antibiotic resistant, while all seedlings were uniformly spectinomycin-sensitive and bleached white when the plastid-transformed lines were used as the pollen donor to wild-type plants. These results confirmed that the pigment deficient phenotype and antibiotic resistance is maternally inherited and not transmitted through pollen, as expected for plastid-encoded traits. Maternal inheritance was further confirmed in T2 and T3 generations of the PTS40 and PTS38 lines, confirming that there is no active copy of the *PDS* transgenes in the nuclear genome.

Although PTS38 and PTS40 T1 seedling cotyledons were uniformly pigment deficient, the first true leaves emerged as partly green and subsequent leaves were uniformly green. However, the pigment deficient phenotype of PTS40 seedlings is stronger and persists longer than in the PTS38 line, as evidenced by faster greening of the PTS38 first true leaves (Fig. 2C). Interestingly, when PTS38 and PTS40 T1 plants were grown in tissue culture to the 4- to 5-leaf stage and green plants were then transferred back to soil, the chlorophyll-deficient phenotype of newly emerged leaves quickly returned (Fig. 1D). These results indicate that the pigment deficiency is subject to developmental timing.

### Chloroplast division is blocked in a tissue-specific manner in chlorophyll deficient lines

To gain insight into possible defects in plastid development that could help potentiate *PDS* gene silencing, plastid morphology in leaves from T1 plants grown in soil was examined by confocal microscopy. Mesophyll cells of wild-type plants and PTS39 lines have characteristic large chloroplasts (average size ∼5 *µ*M) arranged along the periphery of each cell. Chloroplasts appeared fully developed and densely packed, side-by-side. In contrast, PTS38 and PTS40 transplastomic lines contain smaller (average size ∼2.5 *µ*M) plastids, mostly along the periphery of the cell (Supplemental Fig. S2). Interestingly, many plastids appear to be closely associated as pairs, especially visible in the PTS40 line, suggesting a block in plastid development shortly after plastid division. In contrast to the large chloroplasts present in mesophyll cells, the plastids in epidermal cells were observed to be relatively small (∼3 *µ*M average size) and loosely arranged along the cell periphery in wild type, and all transplastomic lines (Supplemental Fig. S2). These results suggested that the *PDS* gene knockdown may not occur in epidermal cells of the plastid transformed lines, allowing some chlorophyll to accumulate in those cells, resulting in the yellow appearance of PTS38 and PTS40 lines. Moreover, although a block in plastid division is apparent, it is unclear if this can contribute to leakage of plastid transcripts into the cytoplasm.

### RT-qPCR confirms knockdown of PDS mRNA in plastid transformed lines

**Figure 3.**
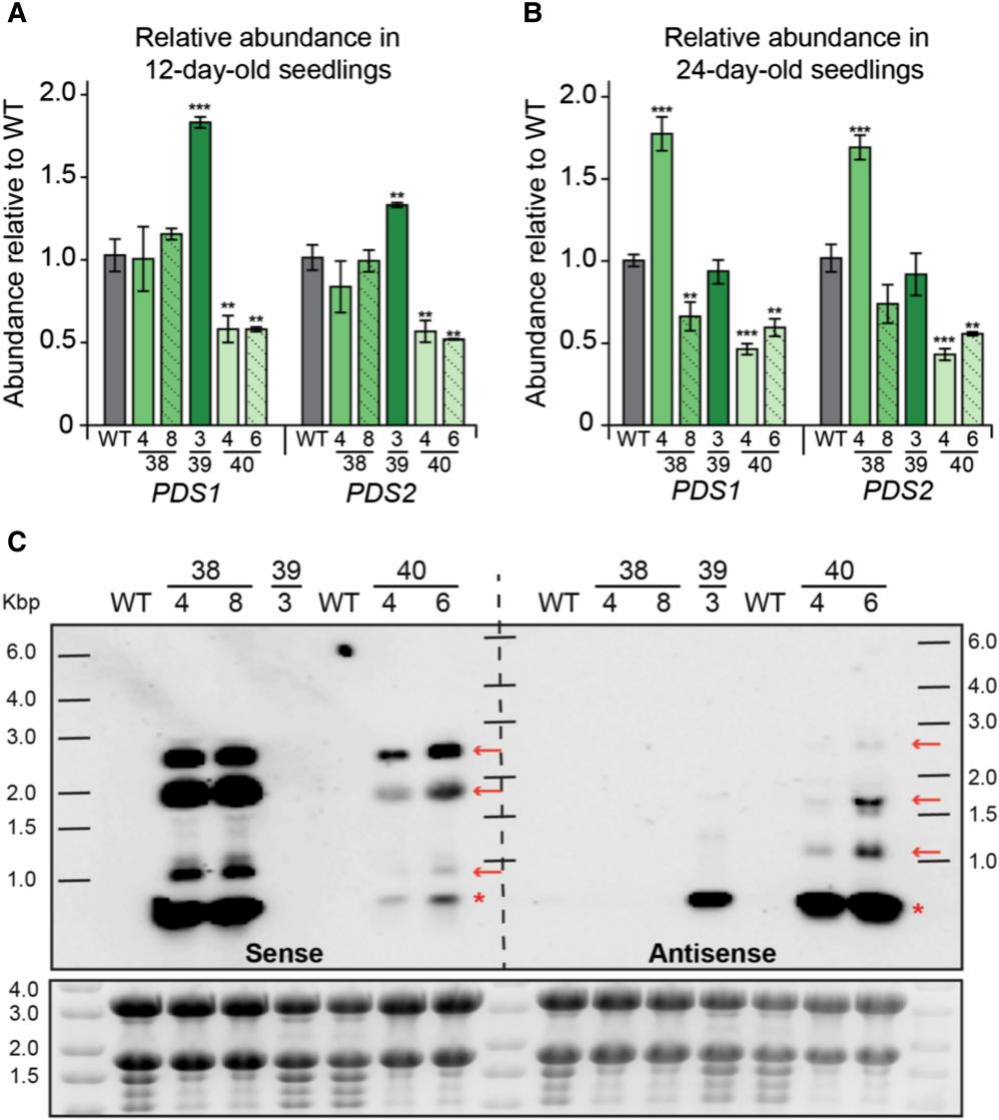
Abundance and expression pattern of nuclear- and plastid-encoded PDS genes. **A, B)** Relative abundance of nuclear-encoded *PDS1* and *PDS2*, as measured by RT-qPCR, in 12-day-old **A**) and 24-day-old **B**) seedlings from WT and PTS38, PTS39, and PTS40 transplastomic lines. All RNA expression values are relative to WT and normalized to the housekeeping gene, *Actin*. Error bars represent SEM. *, **, *** denotes *P* values < 0.05, 0.01, 0.001, respectively, Student's *t*-test. **C)** RNA gel blot with WT and independent transplastomic events of PTS38, PTS39, and PTS40 lines. Membrane was either blotted with a probe recognizing the sense strand of *PDS1* (left) or antisense strand of *PDS1* (right). The red arrows represent likely readthrough transcripts, while the red asterisk represents the PDS-only transcript. Ladder is the RiboRuler High Range RNA ladder. Gel image before transfer is represented below the blot. WT, wild-type; PTS, plastid transformed lines carrying tobacco PDS transgenes; Kbp, kilobase pair.

To determine whether the pigment deficiency of plastid transformed lines is due to silencing of the nuclear-encoded, cytoplasm-localized *PDS1* and/or *PDS2* mRNAs, we examined the abundance of *PDS1* and *PDS2* in T1 transplastomic and wild-type seedlings using RT-qPCR. To ensure we only examined the nuclear encoded RNAs, and not those produced from the transgene, we used PCR primer pairs that map outside of the plastid-encoded PDS transgene (Supplemental Fig. S1A). In 12-day-old seedlings, when cotyledons of PTS38 and PTS40 lines are uniformly bleached (Fig. 1C), the levels of PDS1 and PDS2 mRNA are significantly reduced in the PTS40 dsRNA lines to ∼50% of wild-type levels, while no change in PDS mRNA was observed in the PTS38 lines (Fig. 3A). This confirms that the bleached phenotype observed in the PTS40 lines is likely due to the knockdown of nuclear-encoded PDS mRNA levels, while the phenotype observed in the PTS38 lines is likely due to a different mechanism. In agreement with the green phenotype of the PTS39 line, the levels of PDS1 and PDS2 are not decreased, but even slightly increased (Fig. 3A).

The first true leaves of PTS38 and PTS40 seedlings begin to lose their bleaching phenotype and become green later in development (Fig. 2C). In 24-day-old seedlings of the PTS40 line (Fig. 3B), the mRNA levels of the nuclear-encoded PDS1 and PDS2 remain reduced compared to wild-type, indicating that reversion of the bleaching phenotype is independent of PDS mRNA levels and may suggest additional levels of PDS regulation such as translational control. In contrast, the PDS mRNA levels in the PTS38 lines appear more variable at this later developmental stage (Fig. 3B).

### RNA gel blot analysis confirms high-level expression of plastid-encoded PDS transcripts

Previous RNA gel blot studies showed high-level dsRNA accumulation in plastids, though the relative accumulation of sense and antisense strands expressed from convergent promoters was not examined (Zhang et al. 2015). Using oligonucleotide probes from within the *PDS1* plastid transgene fragment (Fig. 1), accumulation of plastid-encoded *PDS1* sense or antisense (Fig. 3C) RNA from the transplastomic lines was examined. The PTS38 line accumulates very high levels of sense strand transcripts of the expected ∼294-nt *PDS1* fragment size and several higher molecular weight transcripts, which are likely due to inefficient transcriptional termination, as is often observed from plastid genes (Bock 2015). The PTS40 line accumulates similar sized sense-strand transcripts as the PTS38 line, albeit at much lower levels. Interestingly, the PTS40 line accumulates higher amounts of the *PDS1* ∼294-nt fragment antisense transcript, suggesting that unpaired sense and antisense transcripts likely exist in this line, possibly with different levels of RNA stability, in addition to dsRNA. Readthrough transcripts also accumulate to low levels in this line, also suggesting a difference in mRNA stability between sense and antisense transcripts in the PTS40 line.

In contrast to the other transplastomic lines, the PTS39 line accumulates the ∼294-nt sense-strand *PDS1* fragment transcript at lower levels than the PTS38 and PTS40 lines, and readthrough transcripts are only apparent after long exposures of the R NA gel blot. We speculate that lower levels of readthrough *PDS* transcripts in PTS39 are due to the absence of a suitable stabilizing element in the neighboring plastid genome. Lack of observed *PDS* transcripts in the wild-type samples (Supplemental Fig. S3) was anticipated due to relatively low levels of mRNA expression compared to plastid-encoded dsRNAs that can accumulate up to 0.8% of total cellular RNA (Wu et al. 2022).

### Processing of plastid transgene-encoded small RNA in the cytoplasm

**Figure 4.**
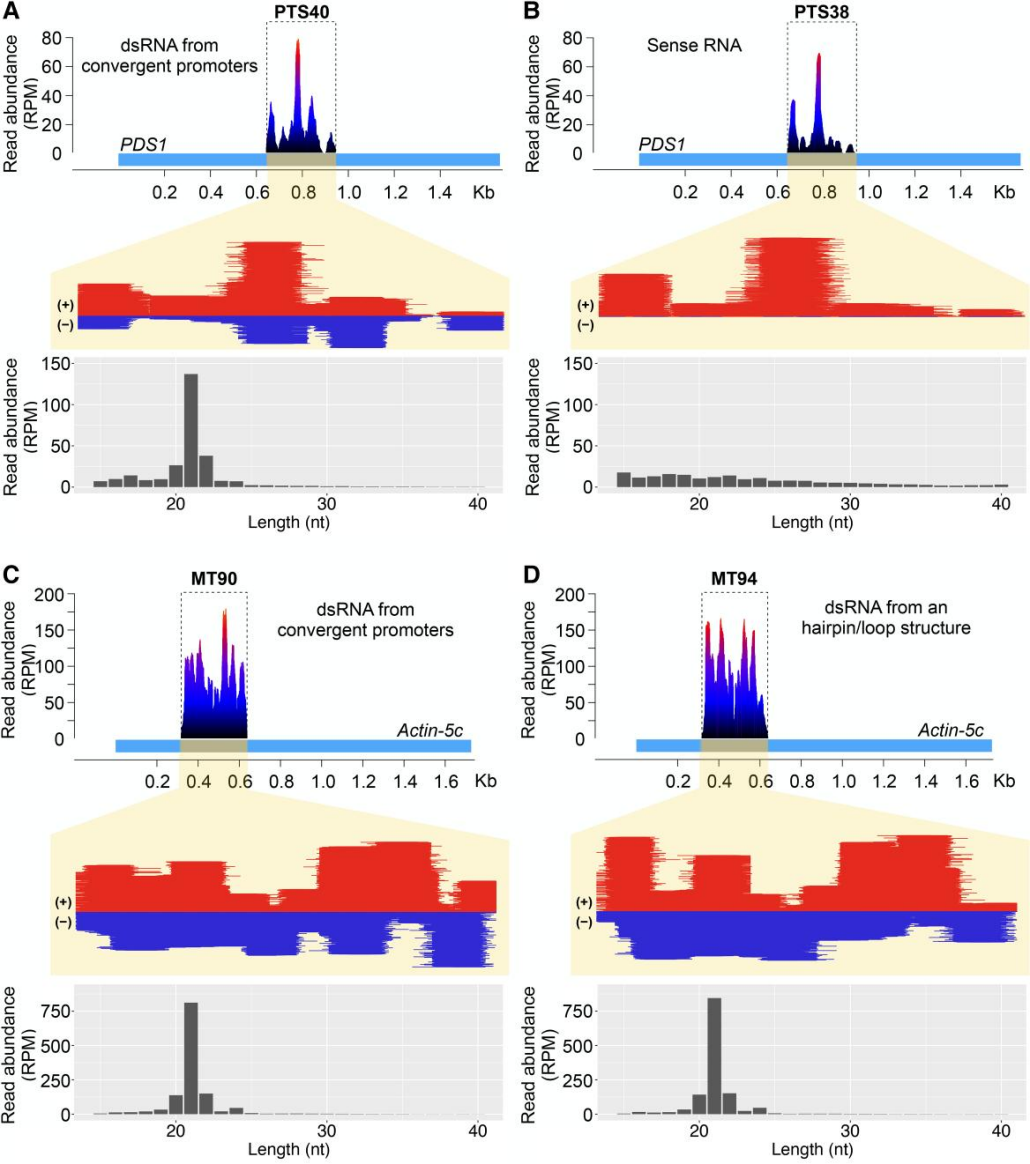
Plastid-expressed dsRNA is processed to 21-nt phasiRNAs. **A**, **B)** Mapping and accumulation of *PDS1* siRNAs (red, sense strand; blue, antisense strand) in the PTS38 and PTS40 transplastomic lines. **A)** Accumulation of predominantly 21-nt siRNAs (below) in the PTS40 line map to both PDS1 sense and antisense strands, whereas **B**) siRNAs in the PTS38 line show no bias in length (below) and map only to the sense strand. The relative location of the *PDS* transgene fragment (gray) in the nuclear-encoded *PDS1* gene (light blue) is represented above the panels. **C, D)** Mapping and accumulation of siRNAs in the MT90 and MT94 lines expressing the *Frankliniella occidentalis Actin-5c* gene from either convergent promoters **C**) or a hairpin/loop RNA **D**) transgene. Panels below show the length distribution of reads showing predominantly 21 nt siRNAs. The relative location of the transgene fragment (gray) in the *Actin-5C* gene (light blue) is represented above the panels. The *y* axis indicates the distribution of read abundance normalized in RPM. PTS, plastid transformed lines carrying tobacco *PDS* transgenes; MT, plastid transformed lines carrying genes from *F. occidentalis*; dsRNAsiRNA, short interfering RNA.

Reduction of nuclear-encoded *PDS* mRNA levels via plastid transgenic RNAs suggests that the latter can enter the PTGS pathway, which is characterized by the presence of 21- or 22-nt siRNAs. Since previous reports of chloroplast-transformed plants carrying dsRNA transgenes did not observe siRNA accumulation by R NA gel blot analysis (Zhang et al. 2015; Bally et al. 2016), we used the higher-resolution technique of small RNA deep sequencing of the 12-day old transplastomic and wild-type seedlings derived from self-pollinated plants. As expected, no siRNAs mapping to either *PDS1* or *PDS2* nuclear genes were detected in wild-type seedlings (Table 1). In contrast, abundant siRNAs (total ∼180 to 280 reads per million, RPM) that match *PDS* were observed in all PTS38 and PTS40 (bleached) lines (Fig. 4 and Table 1). Interestingly, the (green) PTS39 line accumulated detectable siRNA reads, though to a much lower degree (∼20 RPM; Table 1 and Supplemental Fig. S4). Inspection of the siRNA sequences in all lines indicates that none mapped to polymorphic *PDS2* sequences (Supplemental Fig. S5), and no secondary siRNAs derived from the nuclear-encoded *PDS* genes beyond the 294-nt plastid *PDS* transgene region were observed, suggesting that the siRNAs derive directly from plastid transcripts.

**Table 1.** Plant lines, phenotype, and siRNA characterization

Plant line	Expression strategy	Target gene	Phenotype	RPM	Major RNA length	Phase score
PTS38-4	Sense RNA	*PDS*	Bleached	198.0	15	-
PTS38-8	Sense RNA	*PDS*	Bleached	179.8	19	-
PTS39-3	Antisense RNA	*PDS*	Green	19.6	21	17.3
PTS40-4	Convergent promoter	*PDS*	Bleached	280.5	21	35
PTS40-6	Convergent promoter	*PDS*	Bleached	260.2	21	35.3
MT90-2	Convergent promoter	*Actin-5c*	Green	691.7	21	39.7
MT90-5	Convergent promoter	*Actin-5c*	Green	1,295.4	21	61.7
MT91-2	Convergent promoter	*SNF7*	Green	71.1	21	6.5
MT91-4	Convergent promoter	*SNF7*	Green	106.8	21	10.2
MT94-1	Hairpin/loop RNA	*Actin-5c*	Green	832.4	21	57.7
MT94-2	Hairpin/loop RNA	*Actin-5c*	Green	1,331.6	21	51
MT95-1	Hairpin/loop RNA	*SNF7*	Green	186.4	21	52.8
MT95-2	Hairpin/loop RNA	*SNF7*	Green	145.6	21	27.4
WT	N/A	N/A	Green	-	-	-

A dash indicates either no reads were detected, or no phasing score was observed.

WT, wild type; N/A, not applicable.

To provide additional insights into the mechanism of siRNA biogenesis from the plastid transgenes, we examined their size distribution, strandedness, and potential “phasing”. siRNA phasing is an indication of recursive Dicer processing and may be amplified via an RNA-dependent RNA polymerase (RDR protein) acting in the cytoplasm (Liu et al. 2020). For the PTS40 dsRNA lines, siRNAs map abundantly to both strands of the *PDS1* transgene and 21-nt siRNAs make up the vast majority of reads across both strands (Fig. 4A). The phasing analysis for dsRNA PTS40 lines indicated a high phasing score of ∼35 (Table 1). These results indicate that Dicer-like processing resulting in phasiRNAs occurs from plastid-transgene RNAs, and such processing should localize in the cytoplasm of transplastomic plants.

Abundant siRNAs were also mapped to *PDS1* in the transplastomic PTS38 sense strand lines (Fig. 4B). However, in contrast to the PTS40 dsRNA lines, PTS38-derived siRNAs mapped exclusively to the sense strand of the *PDS1* gene, have a broad size range distribution with no discernible size peak and were not phased. These latter results suggest PTS38-derived siRNAs may result from transcript degradation in the cytoplasm or the chloroplast, rather than Dicer-dependent cleavage. Taken together with the RT-qPCR results that indicate no change of nuclear-encoded PDS mRNAs at this 12-day old time point (Fig. 3) and no apparent phasiRNAs, these data suggest that the bleaching phenotype observed in the PTS38 line is likely due to a different mechanism than in the PTS40 lines, possibly translational inhibition of PDS.

Accumulation of 21-nt phasiRNAs in the PTS40 dsRNA line presumably results from processing of plastid-derived transcripts in the cytoplasm after RNA movement by unknown mechanisms. To rule out previously uncharacterized RNA processing events of transgenic transcripts inside the plastid, we prepared small RNA libraries from purified plastids from the same 12-day old transplastomic T1 seedlings as described above. In all transplastomic lines, plastid-localized small RNAs were abundant with a broad size distribution, but no clear peak at 21-nt, and no phasiRNAs were observed (Supplemental Fig. S6). These results confirm that processing of plastid transcripts to phasiRNAs occurred in the cytoplasm and not in the organelle. Multiple reports have previously described stable small RNA footprints in plastids that are protected from degradation by RNA-binding proteins (reviewed in Barkan and Small 2014); however, we did not attempt to identify those RNAs of no apparent function in this work.

### Processing of plastid transgene-derived dsRNA to 21-nt phasiRNAs is a general phenomenon

The finding of abundant 21-nt phasiRNAs derived from the plastid *PDS1* raised the question of whether a nuclear-encoded complementary target RNA is required for processing into siRNAs. To examine this question, we utilized transplastomic tobacco lines that express dsRNA against non-plant (insect) targets, from either 2 convergent plastid promoters identical to the *PDS* dsRNA transgene (MT90 and MT91) or via a single chloroplast promoter driving a hairpin/loop dsRNA construct (MT94 and MT95) (Fig. 4 and Supplemental Fig. S7). The constructs were designed to express dsRNA against 2 insect gene targets, *SNF7* and *Actin5C* from *Frankliniella occidentalis*.

We again performed small RNA sequencing of total cellular RNA from 12-day-old transplastomic seedlings. The results were similar for the convergent chloroplast promoters and the hairpin/loop dsRNA constructs, and across 2 independent transplastomic lines tested for each construct (Fig. 4C and D; Table 1). In all cases, siRNAs were abundant and mapped across both DNA strands of *SNF7* and *Actin5C*, and along the entire genic region that comprises the plastid transgenes, with no apparent transitive siRNAs mapping outside of this region. Importantly, the size distribution of the siRNAs showed a strong peak at 21-nt in all events and each event had a high phasing score from ∼27 to 62 (Table 1). These results confirmed that plastid-expressed dsRNA enters the host cytoplasmic RNAi pathway to generate 21-nt phasiRNAs, are derived from the transgene, and arise independent of complementary nuclear-encoded sequences.

## Discussion

The ability of plastid-encoded RNAs to enter the gene silencing pathway in the cytoplasm is a surprising finding. We observed that plastid-encoded PDS RNAs affect nuclear PDS gene silencing via multiple lines of evidence, including accumulation of 21-nt phasiRNAs that derive from Dicer-like processing of dsRNA in the PTS40 and MT lines (Fig. 4). Interestingly, lack of siRNAs spreading to adjacent PDS1 sequences or siRNAs derived from the PDS2 polymorphic regions suggests that plastid-encoded transcripts can be used directly in the cytoplasm as template for Dicer-like 4 cleavage without RDR activity. In support of this conclusion, the processing of plastid transgene-encoded dsRNAs to 21-nt phasiRNAs does not require a plant host-encoded transcript target to enter the RNAi pathway, as evidenced by abundant phasiRNAs derived from the dsRNA plastid transgenes encoding an insect gene fragment with no cognate nuclear-encoded compl ementary partner.

Plant phasiRNA biogenesis typically initiates with an Argonaute (AGO)-catalyzed cleavage of a single-stranded mRNA precursor, followed by conversion to dsRNA by an RDR protein and processing into 21-nt or 24-nt RNA duplexes by a Dicer-like (DCL) protein (Hung and Slotkin 2021; Liu et al. 2020; Fei et al. 2013). However, there are multiple examples of phasiRNAs generated from dsRNA apparently without AGO-directed cleavage, typically from long fold-back precursors (Kakrana et al. 2018; Liang et al. 2023). These “untriggered phasiRNAs” were also observed from our use of convergent promoters to generate dsRNA from the chloroplast to yield phasiRNAs. The mechanism of biogenesis of phasiRNAs without a triggering miRNA should be examined in future work.

In contrast to phased siRNAs observed in the PTS40 lines, the PTS38 line showed pigment deficient plants with no PDS mRNA knockdown via RT-qPCR (Fig. 3) or phasiRNAs by small RNA sequencing (Fig. 4), suggesting a different mechanism of gene silencing in that line. Translational repression of nuclear genes may occur by interfering with translationally active ribosomes via multiple mechanisms in response to accumulation of translatable transcripts at high concentrations (Li et al. 2013) as is observed in the PTS38 line by RNA gel blot (Fig. 3). Somewhat surprisingly, the antisense PDS RNA in the PTS39 line did not induce bleaching. While this line accumulates plastid PDS RNA at easily detectable levels via RNA gel blot (Fig. 3), it only accumulates very low levels of siRNAs, albeit with a weak phasing score (Table 1), suggesting possible differences in efficiency of escape or stability of the RNA in the cytoplasm. Future work will be needed to determine the mechanism acting from plastid-expressed sense and antisense RNAs.

Movement of the plastid transcripts to the cytoplasm is a prerequisite to phasiRNA production and nuclear-encoded *PDS* gene silencing since the PTGS machinery does not exist in plastids (Zhang et al. 2015; Bally et al. 2016). Our results indicate that movement of transcripts out of the plastid compartment must happen commonly, though may be developmentally regulated, as evidenced by the changing nature of the pigment deficient phenotype. The initial triggering events originate in germinating seedlings and last until the first true leaves emerge partly green, and subsequent plant growth in tissue culture looks normal. Completely green and healthy plants in tissue culture again show bleaching of new leaves when plants are transferred to soil. Bleaching of plant leaves in soil persists for several weeks, suggesting a continual escape of plastid-expressed *PDS* transcripts and continual entry into the gene silencing machinery in the cytoplasm.

These results suggest a continual turnover of a subpopulation of plastids during plant development, reminiscent of autophagic turnover of plastids (reviewed in Izumi et al. 2019; Woodson 2019; Izumi and Nakamura 2018; Zhuang and Jiang 2019), perhaps initially in response to limited photosynthetic activity that induces sugar starvation, or may be a consequence of changes in plastid membranes during proplastid development (Pyke 2013) or the conversion to chloroplasts as has been reported in some plant species (Semenova 2018, 2021). We speculate that plastid redox or other signals accumulating during these developmental stages may trigger plastid autophagy, liberating their contents to the cytoplasm *en route* to the vacuole by unknown means. During this process, highly expressed plastid RNAs are apparently stable enough to be captured by the PTGS apparatus located in the cytoplasm. However, the differences observed in siRNA accumulation levels between the transplastomic lines indicate that the efficiency of escape from the plastids may depend on multiple factors and can be enhanced.

Movement of plastid-derived transcripts to the cytoplasm occurred in the absence of any specific treatments designed to facilitate the process. Our observations are different from previous reports of “escape” of DNA from mitochondria or chloroplasts in which a strong selection is required to identify rare transfer of a chloroplast transgene to the nucleus (Thorsness and Weber 1996; Bock 2017; Huang et al. 2003; Bock and Timmis 2008). Likewise, our work contrasts with a report that utilized paraquat herbicide or bacterial infection to catalyze reactive oxygen species that disrupt chloroplast membranes, thus allowing leakage of a chloroplast-localized fluorescent protein to the cytoplasm (Kwon et al. 2013). It will be interesting to determine if a chloroplast-expressed transgenic protein can also move out of the chloroplast to catalyze effects in other cellular compartments or provide for useful traits thought previously not to be amenable to chloroplast transformation technology.

While the precise molecular mechanisms leading to small RNA production from plastid transgenes and activity in the cytoplasm against a nuclear-encoded gene will require additional research to fully understand, our results have implications for expanding the range of targets available for plastid engineering. Recent studies indicate that transplastomic plants accumulate high levels of long unprocessed dsRNA and are effective against multiple insect pests. In each of these reports, no apparent small RNA production from chloroplast transgenes was observed via RNA gel blots, leading the authors to conclude that DCL proteins and the rest of the RNAi machinery are absent from plastids (Zhang et al. 2015; Bally et al. 2016). Our data suggest that the lack of processing of long dsRNA to siRNA in plastids may not be a limitation for controlling plant pathogens that preferentially take up siRNAs or do not digest plant tissues, for example, viral, nematode and some fungal and oomycete pathogens (Liu et al. 2019; Jaubert-Possamai et al. 2019; Qiao et al. 2021; Taliansky et al. 2021).

The ability to silence plant nuclear genes from the plastid enables engineering of an array of traits previously unavailable to plastid transformation technology, including metabolic processes, biomass, and grain yield (Mansoor et al. 2006; Kamthan et al. 2015; Saurabh et al. 2014). The results reported here present an additional potential pathway for chloroplast-to-nucleus signaling (reviewed in Jan et al. 2022; Liebers et al. 2022).

## Materials and methods

### Construction of plastid transformation vectors

The *PDS1* gene fragment was synthesized and cloned as a KpnI/SbfI DNA fragment between the convergent P*rrn* promoters in vector pJZ199 (Zhang et al. 2015). In pPTS38, the *E. coli rrnB* terminator fragment (T*rrn*; Zhang et al. 2015) was synthesized and cloned as a KpnI/NotI fragment to replace one of the P*rrn* promoters, to create the sense *PDS* construct. In pPTS39, the *rrnB* terminator was cloned as an SbfI/SalI fragment to replace the opposite P*rrn* promoter, creating the antisense *PDS* construct. The *PDS* transgenes (sequences in Supplemental Table S1) were cloned next to a chimeric *aadA* spectinomycin resistance gene driven by *Chlamydomonas reinhardtii* chloroplast *psbA* gene promoter and *rbcL* gene 3′-untranslated region. The *PDS* and *aadA* transgenes are flanked by regions of identity (∼1,950 nt and ∼670 nts) to the tobacco (*N. tabacum* cv Petit Havana) chloroplast genome, resulting in integration of both transgenes between the resident *trnfM* and *trnG* chloroplast genes in plastid transformed plants.

### Plant growth, transformation, and selection of chloroplast transformants

Tobacco (*N. tabacum* cv Petit Havana) plants were grown aseptically from seedlings on MS agar medium for ∼4 weeks at 28 °C under a 16 h light/8 h dark photoperiod (using 4500 K cool fluorescent bulbs, dimmed to ∼50 *µ*E). Young leaves were harvested for particle bombardment, placed abaxial side up and bombarded using the BioRad PDS1000 He gun according to standard procedures (Maliga and Svab 2010). Transplastomic events were selected by growth on 500 mg/L spectinomycin. Primary transformants typically arise as shoots on this medium; young leaf tissue from shoots is dissected and used for a second and subsequently repeated for a third round of plant regeneration on selective medium to ensure homoplasmy of the plastid transformed lines. Plastid transformants were confirmed by PCR-sequencing of amplification products to confirm the entire transgenic insert sequence, including correct junctional sequences with nontransformed wild-type chloroplast genome, as expected for homologous recombination in plastids.

Plastid transformed lines were rooted on MS medium containing 500 mg/L spectinomycin and allowed to grow to the 4- to 5-leaf stage before transfer to soil. For reciprocal crosses of plastid transformed lines to wild-type plants, flowers were emasculated by hand and manually pollinated and then individually bagged until seed pods are mature.

Seeds from wild-type and plastid-transformed plants were surface sterilized using 10% v/v Clorox solution with a few drops of neat Tween-20 for 10 min with shaking. After sterilization, seeds were washed with at least 4 changes of excess sterile water and sown on agar medium at 24 °C with 16 h light for 12 days prior to harvest. Two events per construct were grown for isolation of whole-cell and chloroplast-enriched RNA fractions.

### Confocal microscopy

For imaging of leaf tissues, samples were syringe infiltrated with water before microscopic analysis and imaged on a Leica SP8-X (Leica Microsystems, Wetzlar Germany) with a water immersion 63 × HC Plan Apochromat CS2 objective lens (numerical aperture 1.2). Cell wall autofluorescence was visualized using 649 nm (WLL) excitation (laser power at 6%, AOBS 38.73%), 658 to 768 nm emission and photomultiplier tube detector (PMT). PMT and HyD detector gain settings were adjusted for qualitative results and optimized for each sample to use the full dynamic range of the 8-bit images with an offset of 0. Additional image acquisition settings included unidirectional scan and speed 400 Hz, 0 degree rotation, Zoom 1, Pinhole 1 Airy Unity (111.5 *μ*m) with 4 line averages. Three-dimensional (3D) image stacks were acquired with a pinhole of 1 airy unit, 512 × 512 pixel images, field-of-view of 184.52 *µ*m^2^ and 0.9 *µ*m z-interval. Z-stacks were rendered as a 3D maximum intensity projection. Z-stacks of the epidermal and mesophyll layers for each treatment were extracted and rendered as a 3D maximum intensity projection.

### RT-qPCR of PDS1 and PDS2 levels in transgenic plants

To examine levels of *PDS1* and *PDS2* in PTS38, 39, and 40, seedlings were collected 12 days and 4 weeks after sowing on MS media and directly frozen in liquid nitrogen. Tissue was then pulverized in liquid nitrogen and transferred to TRIzol Reagent (Invitrogen, Carlsbad, CA) and RNA was isolated according to the manufacturer's instructions. RNA was then treated with RNase-free DNase (Qiagen, Valencia, CA) for 25 min at room temperature, ethanol precipitated and resuspended in nuclease-free water. Reverse transcription (RT) was performed using Advantage RT-for-PCR kit (Takara, San Jose, CA) following the manufacturer's instructions with 500 ng total RNA as input and 20 *µ*M oligo (dT)_18_. cDNA was diluted 1:10 in nuclease-free water for RT-qPCR.

RT-qPCR was performed using 2 × PowerUp SYBR Green Master Mix (Applied Biosystems, Carlsbad, CA) as follows per well: 5 *µ*L 2 × PowerUp SYBR Green Master Mix, 0.75 *µ*L cDNA (diluted 1:10), 1.25 *µ*L nuclease-free water, and 3 *µ*L combined 1.5 *µ*M forward and reverse primers. All RT-qPCR reactions were performed in 3 technical replicates and all primers were tested for non-specific amplification using water and specificity using genomic DNA and analysis of melt curves. Technical replicates refer to triplicates of the same sample to provide an estimate of precision and allows for outlier detection and experimental variation. Two biological replicates were included for each line. Biological replicates refers to seedlings grown on MS plates as described above where half of each MS plate was collected for biological replicates. All reactions were run using the following program: 95 °C for 10 min; 40 cycles of 95 °C 30 s, 55 °C 30 s, 72 °C 30 s. Melt curves were generated by heating the final PCR 1.6 °C/s to 95 °C for 15 s, decreasing the temperature to 60 °C at 1.6 °C/s and slowly increasing back to 95 °C at 0.1 °C/s. All primers are listed in Supplemental Table S2. Results were first normalized to the housekeeping gene *Actin* (dC_T_) and then normalized to wild-type (ddC_T_). Relative ddC_T_ value was calculated as 2^−(ddCt)^. Error bars represent standard error of the mean (SEM).

### RNA gel blot analysis

For the denaturing agarose RNA gel blot, 20 *µ*g of DNase-treated total RNA was diluted to a final volume of 24.5 *µ*L in nuclease-free water. 2 × NorthernMax Formaldehyde Load Dye (Invitrogen, Carlsbad, CA) was added to the RNA samples and 10 *µ*L RiboRuler High Range RNA Ladder (ThermoScientific, Carlsbad, CA) and denatured for 15 min at 65 °C. RNA was immediately placed on ice, and 1 *µ*L ethidium bromide (20 mg/mL) was added and mixed well. Probes were prepared using the Biotin 3′ End DNA Labeling Kit (ThermoScientific, Rockford, IL) as described by the manufacturer's instructions. Probes were synthesized by IDT (Newark, NJ) and are listed in Supplemental Table S2.

To make the denaturing agarose gel, UltraPure agarose (Invitrogen, Carlsbad, CA) was dissolved in water, allowed to cool to ∼60 °C and 10 × NorthernMax Denaturing Gel Buffer (Invitrogen, Carlsbad, CA) (pre-warmed to 60 °C) was added to have 1.2% w/v agarose gel. Ten micrograms of RNA was loaded into each well (each sample was split between 2 wells) and run in 1 × MOPS Electrophoresis Buffer (Fisher Bioreagents) for 2.5 h at 95 V. The gel was imaged and washed 2 × 15 min in nuclease-free water then 2 × 15 min in 10 × UltraPure SSC Buffer (Invitrogen, Carlsbad, CA) with gentle shaking. Samples were transferred to BrightStart—Plus membrane (Invitrogen, Carlsbad, CA) membrane using capillary action in 10 × UltraPure SSC Buffer overnight. The next day, RNA was crosslinked to the membrane using Stratagene UV Stratalinker 2,400 and incubated for 30 min in pre-warmed NorthernMax Prehyb/Hyb Buffer (Invitrogen, Carlsbad, CA) at 42 °C in a hybridization oven with rotation. Twenty microliters of biotinylated probes were added to Prehyb/Hyb solution and incubated overnight at 42 °C with rotation. The next day, the probe was removed and the membranes were washed in 2 × SSC, 0.5% SDS wash solution 2 × 30 min. The membranes were then treated with the Chemiluminescent Nucleic Acid Detection Module (ThermoScientific, Carlsbad, CA) for detection, according to the manufacturer's instructions. Membranes were visualized using BioRad ChemiDoc XRS + .

### DNA gel blot analysis of plastid transformed lines

Leaves from ∼6- to 8-week-old plants grown aseptically in tissue culture were used for total cellular DNA isolation using the DNAZol reagent (ThermoFisher) according to manufacturer's instructions. Three micrograms of total cellular DNA is digested by BglII restriction enzyme, electrophoresed in an agarose gel, and digested DNA transferred to nylon membrane according to standard procedures. The probe for DNA hybridization was synthesized using DIG probe kit (Sigma-Aldrich) and used for overnight hybridization at 55 °C. Washing and processing of the blot was performed according to standard procedures.

### Chloroplast-enriched RNA fraction isolation, library construction, and sequencing

To isolate the chloroplast-enriched RNA fraction, we isolated chloroplasts from fresh leaves using the Minute Chloroplast Isolation Kit (Invent Biotechnologies, Plymouth, MA), following the manufacturer's instructions. To isolate enough RNA, per sample, we isolated chloroplast from 2 preps of 250 mg of tobacco leaf. We isolated the chloroplast-enriched RNA fraction with the TRI Reagent (Sigma-Aldrich, St. Louis, MI) following the manufacturer's instructions. To assess the purity of chloroplast-enriched RNA, between the whole-cell and chloroplast-enriched RNA fractions, we compared the abundance of 2 highly expressed miRNAs, miR156 and miR168, in tobacco leaves and observed that chloroplast RNA is enriched at 90% (Supplemental Table S3).

### Small library construction, sequencing, and bioinformatic analysis

To collect the whole-cell RNA fraction, plantlets for each line were harvested in 3 or 5 replicates and, after dissection, samples were immediately frozen in liquid nitrogen and kept at −80 °C before RNA isolation. For replicates, seedlings were grown on MS plates as described above and an entire plate was collected for each biological replicate. sRNA libraries were constructed using the RealSeq-AC miRNA Library Kit for Illumina sequencing (Somagenics, Santa Cruz, CA) using an input of 150 ng total RNA and 16 PCR amplification cycles. We size-selected sRNA libraries for the end product of ∼150-nt using the SPRIselect Reagent (Beckman Coulter Life Sciences, Indianapolis, IN) magnetic beads. All libraries were quantified on a DeNovix apparatus (Wilmington, DE) using the Qubit dsDNA Assay Kit (Thermo Fisher Scientific, Waltham, MA) and we multiplexed libraries in 10 nM pools. Single-end sequencing was performed with 76 cycles (3 lanes). The sequencing was generated on an Illumina NextSeq 550 instrument (Illumina) at the University of Delaware DNA Sequencing and Genotyping Center.

We used cutadapt v3.4 (Martin 2011) to preprocess sRNA-seq reads, removing the 3′ adapter and discarding trimmed reads shorter than 15-nt or longer to 40-nt. We mapped cleaned reads to *PDS1* (NCBI ID: XM_016610712.1) and *PDS2* (NCBI ID: XM_016642615.1) transcripts using ShortStack v3.8.5 (Johnson et al. 2016) with following parameters: -mismatches 0, -mmap u, -dicermin 15, -dicermax 40, and -mincov 1.0 RPM. We used the ShortStack analysis report to identify a phasiRNA-generating feature over reads mapping each *PDS* gene for each study.

To visualize sRNA mapping to *PDS* genes, we converted mapping files into Bed Graph and Bed files using functions genomecov and bamtobed from bedtools v2.30.0 (Quinlan and Hall 2010), respectively. We used the R package Sushi v1.24.0 (Phanstiel et al. 2014) to represent the position of transgenes over *PDS* genes and to visualize the coverage and read distribution of sRNAs over *PDS1* and *PDS2* genes.

To investigate properties of sRNA mapping to *PDS* genes, we investigated the read length distribution and the distribution of reads over *PDS* genes for constructs with a high Phase Score. The Phase Score refers to the periodicity of siRNAs produced by Dicer-like processing; higher scores indicate more phasing signatures and increasing evidence for true positive phasiRNA accumulation. (i) We first summarized siRNA reads mapping to *PDS* genes into unique tags. We count the total number of reads per length and use the R ggpubr package (Wickham 2016; Kassambara 2020) to draw a bar plot of the read length distribution. For construction with a significant Phase Score, we had assigned the positions within the *PDS* genes to “phasing” bins to one of the 21 arbitrary bins, which repeat in 21-nt cycles (lines). We calculate the abundance of reads in each bin and visualize results on a radar plot. We followed the previously-described method for the detection of phased loci (Axtell 2010).

### Statistical analysis

Statistical analyses were performed as described in each figure legend. Statistical data are provided in Supplemental Table S4.

### Accession numbers

Sequence data of the genes used in this article can be found in Genbank under the following accession numbers: PDS1 (XM_016610712.1) and PDS2 (XM_016642615.1). The sRNA-seq data and processed files are available in NCBI's Sequence Read Archive under BioProject ID PRJNA907077.

## Supplementary Material

koad165_Supplementary_Data

## Supplemental data

The following materials are available in the online version of this article.

**Supplemental Figure S1**. Nuclear-encoded *PDS* gene organization, alignment and fragment insertion into the plastid genome.

**Supplemental Figure S2**. Confocal microscopy indicates small plastids accumulate in PTS38 and PTS40 lines in a cell-type specific manner.

**Supplemental Figure S3**. Lack of detectable nuclear *PDS* mRNA via RNA gel blot of chloroplast transgene RNAs.

**Supplemental Figure S4**. Mapping and abundance of *PDS* siRNAs in the PTS39-3 antisense RNA line.

**Supplemental Figure S5**. siRNAs in the PTS38 and PTS40 lines do not map to the polymorphic regions of the nuclear PDS2 gene.

**Supplemental Figure S6**. Mapping of transgene derived small RNAs in purified chloroplast fractions of PTS38 and PTS40 lines.

**Supplemental Figure S7**. Plastid-expressed dsRNA is processed to 21-nt phasiRNAs.

**Supplemental Table S1**. Sequences of the *PDS* transgenes.

**Supplemental Table S2**. Oligonucleotide probes used for RT-qPCR and RNA gel blot experiments.

**Supplemental Table S3**. Purity of isolated chloroplast fractions as estimated from abundance of contaminating nuclear-encoded miRNAs.

**Supplemental Table S4**. Raw statistical values for Welch 2-sample *t*-test.
