# Supplementary material for: Plastid dsRNA transgenes trigger phased small RNA-based gene silencing of nuclear-encoded genes
Source: Plant Cell. 2023 Jun 13;35(9):3398–412. doi: 10.1093/plcell/koad165 (PMC10473229; doi:10.1093/plcell/koad165)
Supplement: koad165_Supplementary_Data [file koad165_supplementary_data.zip › All supplemental Figs.pdf]

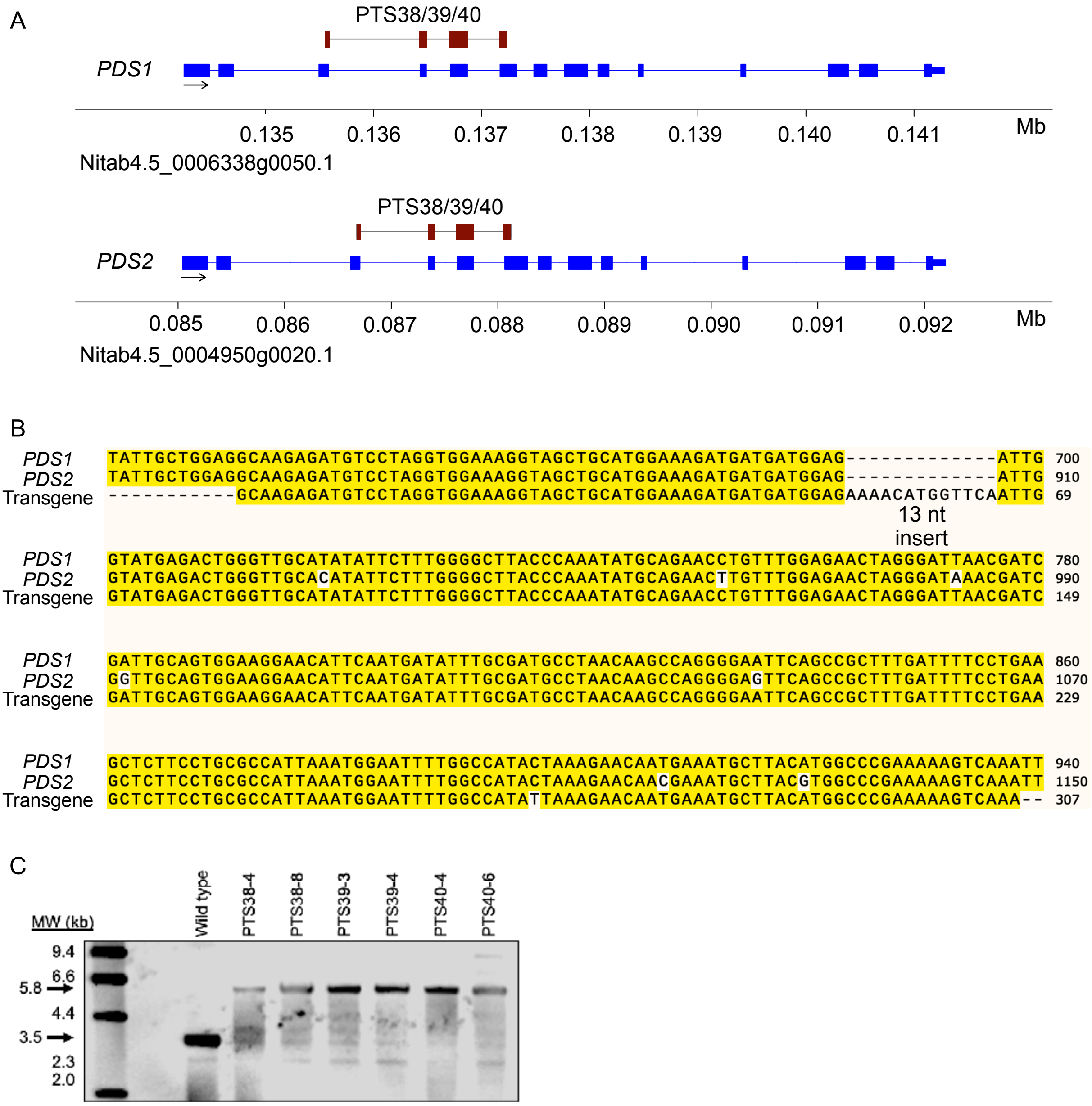

Supplemental Figure S1. Nuclear-encoded PDS gene organization, alignment and fragment insertion into the plastid genome. Supports Figure 1.

(A) Intron (blue lines) and exon (blue box) structure of the two nuclear-encoded *PDS* genes and cDNA fragment (red boxes) used for insertion into the plastid genome. (B) Alignment of *PDS1*, *PDS2* and the PTS transgene cDNA fragment. A 13-nucleotide sequence was inserted in the transgene fragment to discriminate plastid transgenes from the nuclear *PDS* genes. (C) A DNA gel blot showing the integration of the *PDS1* fragment and *aadA* transgenes at the expected location in the plastid genome. BglII restriction enzyme digest results in a ~5.8 kb fragment in the transplastomic lines while wild-type plants without transgene insertion carry an ~3.5 kb band. Note the lack of the ~3.5 kb band in transplastomic lines, indicating they are homoplasmic for the transgene insertion. Note that the homoplasmic line PTS39-4 is not described in the manuscript due to loss of seeds during storage. *PDS1/2*, *Phytoene desaturase* gene 1 and 2*;* MW, Molecular weight.

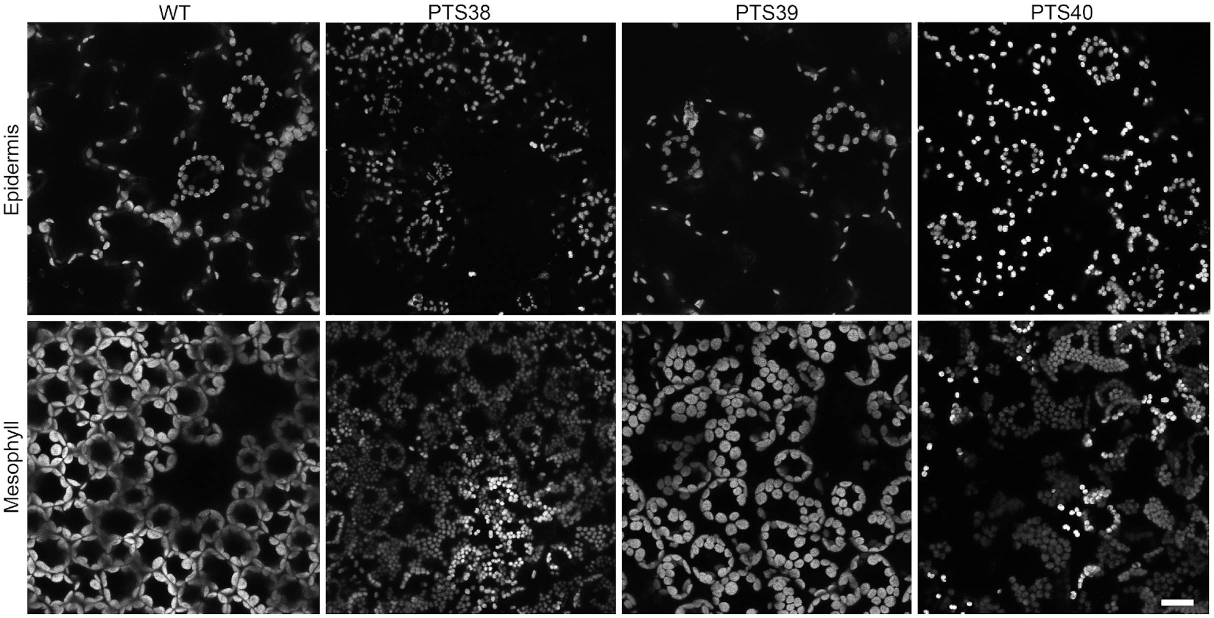

Supplemental Figure S2. Confocal microscopy indicates small plastids accumulate in PTS38 and PTS40 lines in a cell-type specific manner. Supports Figure 2.

The confocal images show the size, development stage and location of plastids in mesophyll and epidermis cells in leaves of wild-type, PTS38, PTS39 and PTS40 lines. The scale bar at bottom right equals 20 µm and is consistent across images, showing smaller plastids in the PTS38 and PTS40 line mesophyll cells compared to wild-type (WT) and PTS39 lines. Also note apparent paired plastids that are especially prevalent in the PTS40 images. Dense packing of cells especially in mesophyll images of PTS38 and PTS40 lines is due to slightly disordered leaf morphology in those pigment deficient lines and the expanded depth of field after confocal Z-stack image processing. PTS*,* Plastid transformed lines carrying tobacco *PDS* transgenes.

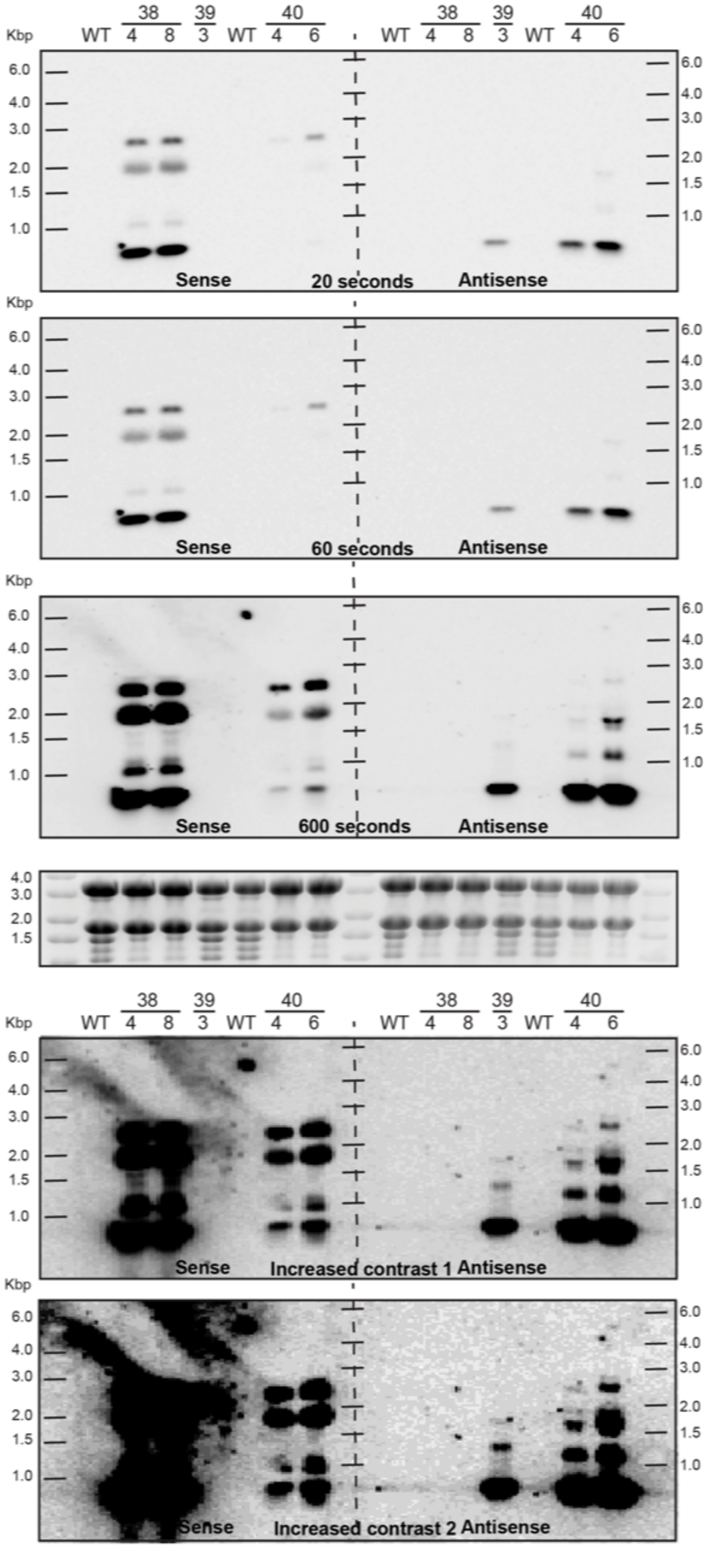

Supplemental Figure S3. Lack of detectable nuclear PDS mRNA via RNA gel blot of chloroplast transgene RNAs. Supports Figure 3.

The chemiluminescent reaction used to visualize hybridizing bands on the RNA gel blot shown in Figure 3 was developed for different lengths of time to enable accurate visualization and detection of both strong and weak signals. The contrast of the digital image was then increased significantly (top blot) and to maximum contrast (bottom blot) to further reveal any visible signal in the wild-type (WT) lanes. Note that a signal for the nuclear *PDS* gene is not evident even at highest signal intensity and contrast, confirming its low expression relative to the plastid *PDS* transgene. *PDS1, 2*, *Phytoene desaturase* gene 1 and 2.

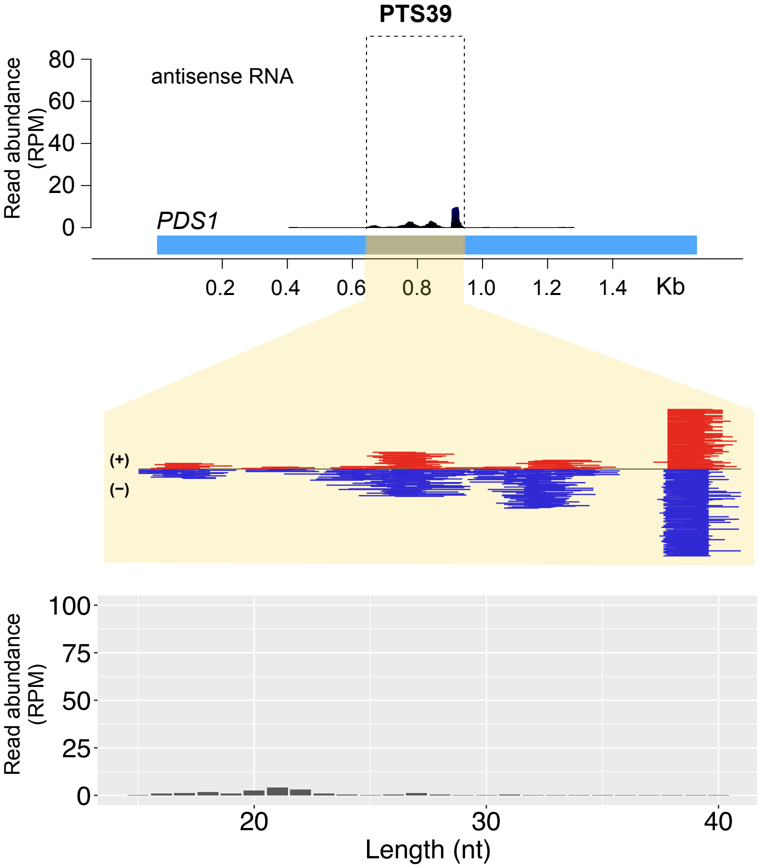

Supplemental Figure S4. Mapping and abundance of PDS siRNAs in the PTS39-3 antisense RNA line. Supports Figure 4.

Low abundance siRNAs accumulate on both sense and antisense strands in the PTS39-3 line. Although abundance is low, the predominant read length is 21 nt (below) and a moderate phasing score is observed for these siRNAs. The relative location of the transgene fragment (gray) in the *PDS* gene (light blue) are represented above the panels. The y-axis indicates the distribution of read abundance normalized in reads per million (RPM). PTS*,* Plastid transformed lines carrying tobacco *PDS* transgenes; *PDS1*, *Phytoene desaturase* gene 1; nt, nucleotide; Kb, kilobase pairs; siRNA, short interfering RNA.

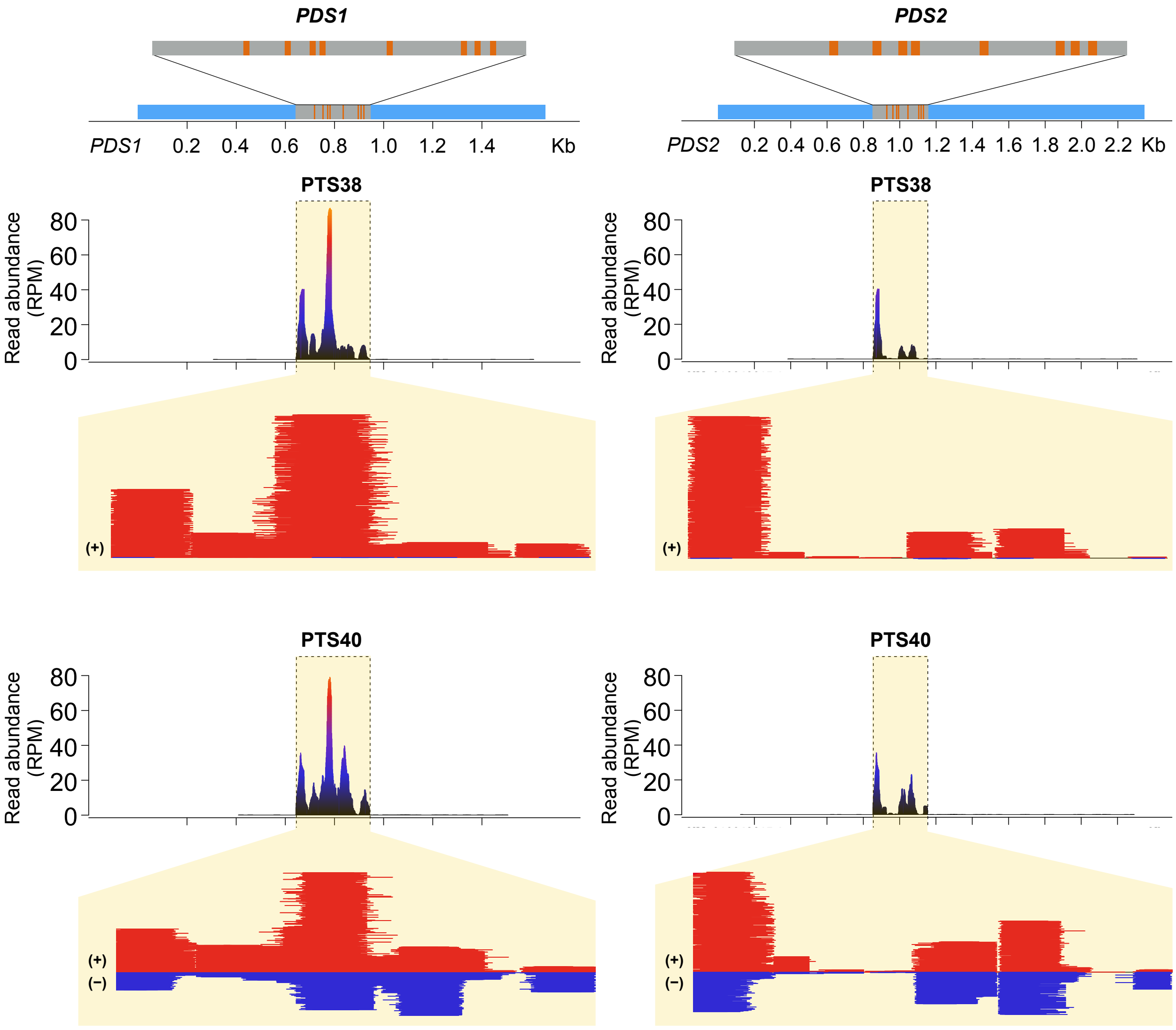

Supplemental Figure S5 siRNAs in the PTS38 and PTS40 lines do not map to the polymorphic regions of the nuclear PDS2 gene. Supports Figure 4.

The figure shows the location of nucleotide polymorphisms (orange bars) between *PDS1* (left) and *PDS2* (right) gene regions. siRNA reads distributed to each gene are shown for PTS38 (top) and PTS40 (bottom) lines. Note the absence of siRNAs mapping to the 7 polymorphic positions of the *PDS2* gene. The y-axis indicates the distribution of read abundance normalized in reads per million (RPM). (+) refers to the sense strand and (-) refers to the antisense strand of the PDS gene fragment. PTS, Plastid transformed lines carrying tobacco *PDS* transgenes; *PDS1, 2*, *Phytoene desaturase* gene 1 and 2, respectively; nt, nucleotide; Kb, kilobase pairs; siRNA, short interfering RNA.

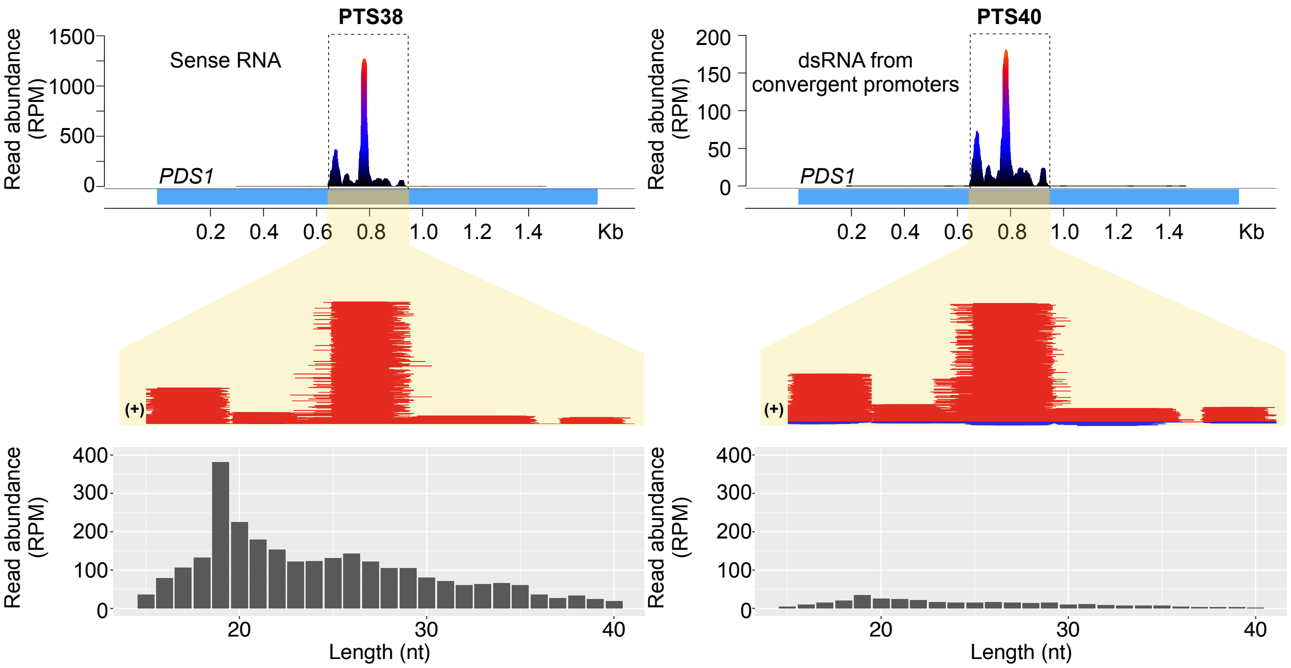

Supplemental Figure S6 Mapping of transgene derived small RNAs in purified chloroplast fractions of PTS38 and PTS40 lines. Supports Figure 4.

Mapping and accumulation of *PDS1* small RNAs (red, sense strand; blue, antisense strand) in the chloroplast fractions of PTS38 (left) and PTS40 (right) transplastomic lines. The relative location of the *PDS* transgene fragment (gray) in the nuclear-encoded *PDS1* gene (light blue) is represented above the panels. Note that the mapping pattern of small RNA reads from the PTS40 transgene differs from the whole-cell RNA fraction because sRNAs accumulate only on the positive strand of the transgene, and neither line accumulates any phasiRNAs in the chloroplast fraction. The y-axis indicates the distribution of read abundance normalized in read per millions (RPM). PTS*,* Plastid transformed lines carrying tobacco *PDS* transgenes; *PDS1*, *Phytoene desaturase* gene 1; dsRNA, double-stranded RNA; nt, nucleotide; phasiRNA, phased short interfering RNA.

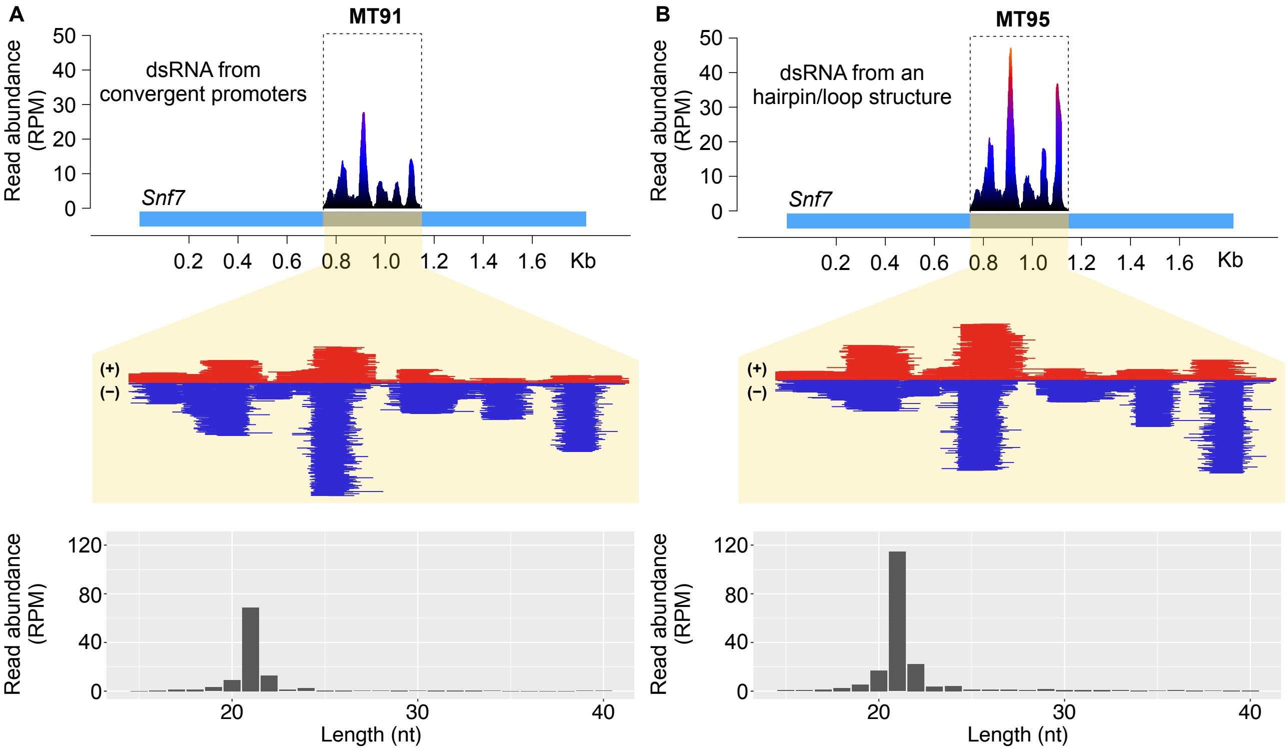

Supplemental Figure S7 Plastid-expressed dsRNA is processed to 21-nt phasiRNAs. Supports Figure 4.

(A, B) Mapping and accumulation of siRNAs in the MT91 and MT95 lines expressing the *Frankliniella occidentalis SNF7* gene from either convergent promoters (A) or a hairpin/loop RNA (B) transgene. Panels below show the length distribution of reads showing predominantly 21 nt siRNAs. The relative location of the transgene fragment in the *Snf7* gene is represented above the panels. The y-axis indicates the distribution of read abundance normalized in reads per million (RPM). MT, Plastid transformed lines carrying genes from *Frankliniella occidentalis*; dsRNA, double-stranded RNA; nt, nucleotide; Kb, kilobase pairs; phasiRNA, phased short interfering RNA; siRNA, short interfering RNA.

Supplementary Table S1. Sequences of the PDS transgenes.

>pPTS38 PDS cassette
CTCGAGGTCGACGGTATCGATAAGCTTGCTCCCCCGCCGTCGTTCAATGAGAATGGATAAGAGGC
TCGTGGGATTGACGTGAGGGGGCAGGGATGGCTATATTTCTGGGAGCGAACTCCGGGCGAATAC
GAAGCGCTTGGATACGCATGCCTGCAGGGCAAGAGATGTCCTAGGTGGAAAGGTAGCTGCATGG
AAAGATGATGATGGAGAAAACATGGTTCAATTGGTATGAGACTGGGTTGCATATATTCTTTGGGGC
TTACCCAAATATGCAGAACCTGTTTGGAGAACTAGGGATTAACGATCGATTGCAGTGGAAGGAACA
TTCAATGATATTTGCGATGCCTAACAAGCCAGGGGAATTCAGCCGCTTTGATTTTCCTGAAGCTCT
TCCTGCGCCATTAAATGGAATTTTGGCCATATTAAAGAACAATGAAATGCTTACATGGCCCGAAAAA
GTCAAATTTGCTGGGTACCATCAAATAAAACGAAAGGCTCAGTCGAAAGACTGGGCCTTTCGTTTT
ATCTGTTGTTTGTCGGTGAACGCTCTCCTGAGTAGGACAAATCCGCCGGGAGCGGATTTGAACGT
TGCGAAGCAACGGCCCGGAGGGTGGCGGGCAGGACGCCCGCCATAAACTGCCAGGCATCAAAT
TAAGCAGAAGGCCATCCTGACGGATGGCCTTTTTGCGTTTCTACGGCCGCCACCGCGGT

> pPTS39 PDS cassette
CTCGAGGTCGAGTAGAAACGCAAAAAGGCCATCCGTCAGGATGGCCTTCTGCTTAATTTGATGCC
TGGCAGTTTATGGCGGGCGTCCTGCCCGCCACCCTCCGGGCCGTTGCTTCGCAACGTTCAAATC
CGCTCCCGGCGGATTTGTCCTACTCAGGAGAGCGTTCACCGACAAACAACAGATAAAACGAAAG
GCCCAGTCTTTCGACTGAGCCTTTCGTTTTATTTGATCCTGCAGGGCAAGAGATGTCCTAGGTGG
AAAGGTAGCTGCATGGAAAGATGATGATGGAGAAAACATGGTTCAATTGGTATGAGACTGGGTTG
CATATATTCTTTGGGGCTTACCCAAATATGCAGAACCTGTTTGGAGAACTAGGGATTAACGATCGAT
TGCAGTGGAAGGAACATTCAATGATATTTGCGATGCCTAACAAGCCAGGGGAATTCAGCCGCTTT
GATTTTCCTGAAGCTCTTCCTGCGCCATTAAATGGAATTTTGGCCATATTAAAGAACAATGAAATGC
TTACATGGCCCGAAAAAGTCAAATTTGCTGGGTACCGAGCTCGTATCCAAGCGCTTCGTATTCGC
CCGGAGTTCGCTCCCAGAAATATAGCCATCCCTGCCCCCTCACGTCAATCCCACGAGCCTCTTAT
CCATTCTCATTGAACGACGGCGGGGGAGCGAATTCCTGCAGCCCGGGGGATCCACTAGTTCTAG
AGCGGCCGCCACCGCGGT

> pPTS40 PDS cassette
CTCGAGGTCGACGGTATCGATAAGCTTGCTCCCCCGCCGTCGTTCAATGAGAATGGATAAGAGGC
TCGTGGGATTGACGTGAGGGGGCAGGGATGGCTATATTTCTGGGAGCGAACTCCGGGCGAATAC
GAAGCGCTTGGATACGCATGCCTGCAGGGCAAGAGATGTCCTAGGTGGAAAGGTAGCTGCATGG
AAAGATGATGATGGAGAAAACATGGTTCAATTGGTATGAGACTGGGTTGCATATATTCTTTGGGGC
TTACCCAAATATGCAGAACCTGTTTGGAGAACTAGGGATTAACGATCGATTGCAGTGGAAGGAACA
TTCAATGATATTTGCGATGCCTAACAAGCCAGGGGAATTCAGCCGCTTTGATTTTCCTGAAGCTCT
TCCTGCGCCATTAAATGGAATTTTGGCCATATTAAAGAACAATGAAATGCTTACATGGCCCGAAAAA
GTCAAATTTGCTGGGTACCGAGCTCGTATCCAAGCGCTTCGTATTCGCCCGGAGTTCGCTCCCAG
AAATATAGCCATCCCTGCCCCCTCACGTCAATCCCACGAGCCTCTTATCCATTCTCATTGAACGAC
GGCGGGGGAGCGAATTCCTGCAGCCCGGGGGATCCACTAGTTCTAGAGCGGCCGCCACCGCG
GT

PTS*,* Plastid transformed lines carrying tobacco *PDS* transgenes; *PDS*, *Phytoene desaturase* gene

Supplemental Table S2. Oligonucleotide probes used for RT-qPCR and RNA gel blot experiments.

**Sequence**	**Name**	**Use**
CCTGAGGTCCTTTTCCAACCA	Actin RT-qPCR Forward	Amplify Actin for RT-qPCR
GGATTCCGGCAGCTTCCATT	Actin RT-qPCR Reverse	Amplify Actin for RT-qPCR
CATTCCGAGGCTTAATTTACCG	Nuclear *PDS1* qRT-PCR Forward	Amplify Nuclear *PDS1* for RT-qPCR
CTTTCAGTTCCCAACGAAGACC	Nuclear *PDS1* qRT-PCR Reverse	Amplify Nuclear *PDS1* for RT-qPCR
TTGGAATTGGTATTTGCACCTG	Nuclear *PDS2* qRT-PCR Forward	Amplify Nuclear *PDS2* for RT-qPCR
TTTTTGCTTTGCTCTGATCTGC	Nuclear *PDS2* qRT-PCR Reverse	Amplify Nuclear *PDS2* for RT-qPCR
GGTGGAAAGGTAGCTGCATGGA	PTS38, 39, 40 Sense Northern blot probe	Detect sense transcription from transgene on RNA gel blot
TCCATGCAGCTACCTTTCCACC	PTS38, 39, 40 Antiense Northern blot probe	Detect antisense transcription from transgene on RNA gel blot

PDS1, 2, *Phytoene desaturase* gene 1 and 2, respectively; RT-qPCR, Reverse transcription quantitative real-time PCR.

Supplemental Table S3. Purity of isolated chloroplast fractions as estimated from abundance of contaminating nuclear-encoded miRNAs.

Construct name	Abundance of miR156			Abundance of miR168		
	Chloroplast fraction (CHL) in RPM	Whole-cell extract (WCE) in RPM	CHL/WCE	Chloroplast fraction (CHL) in RPM	Whole-cell extract (WCE) in RPM	CHL/WCE
PTS38-4	889.5	12,950.3	0.07	325.3	4505.7	0.07
PTS38-8	1197.6	12,151.3	0.10	312.6	3260.2	0.10
PTS39-3	1285.5	23,302.2	0.06	307.6	4592.3	0.07
PTS39-4	802.0	22,191.6	0.04	212.7	5688.2	0.04
PTS40-4	418.9	13,439.8	0.03	194.6	4585.8	0.04
PTS40-6	439.0	13,010.1	0.03	163.7	4177.1	0.04
WT	609.8	18,536.0	0.03	147.0	4367.5	0.03

WT, wild-type.

Supplemental Data Set 1. Statistical analyses were performed as described in figure 3.

Sample 1	Sample 2	t	df	p-value	Significant	Sample 1	Sample 2	t	df	p-value	Significant
PDS1 - Related to Figure 3A											
earlyWT	early38-4	0.10	7.37	9.23E-01	FALSE	lateWT	late38-4	-7.06	6.22	3.43E-04	TRUE
earlyWT	early38-8	-1.23	6.20	2.62E-01	FALSE	lateWT	late38-8	3.60	6.67	9.51E-03	TRUE
earlyWT	early39-3	-7.78	6.00	2.37E-04	TRUE	lateWT	late39-3	0.87	7.36	4.13E-01	FALSE
earlyWT	early40-4	3.51	9.67	5.91E-03	TRUE	lateWT	late40-4	10.99	9.92	7.12E-07	TRUE
earlyWT	early40-6	4.53	5.19	5.66E-03	TRUE	lateWT	late40-6	6.38	3.98	3.14E-03	TRUE
PDS2 - Related to Figure 3B											
earlyWT	early38-4	1.02	7.25	3.41E-01	FALSE	lateWT	late38-4	-6.01	9.87	1.37E-04	TRUE
earlyWT	early38-8	-1.23	6.20	2.62E-01	FALSE	lateWT	late38-8	3.60	6.67	9.51E-03	TRUE
earlyWT	early39-3	-4.10	5.33	8.12E-03	TRUE	lateWT	late39-3	0.64	8.61	5.36E-01	FALSE
earlyWT	early40-4	4.43	9.81	1.33E-03	TRUE	lateWT	late40-4	6.44	6.75	4.10E-04	TRUE
earlyWT	early40-6	6.50	5.03	1.27E-03	TRUE	lateWT	late40-6	5.45	5.18	2.53E-03	TRUE
