# Supplementary material for: Plastid dsRNA transgenes trigger phased small RNA-based gene silencing of nuclear-encoded genes
Source: Plant Cell. 2023 Jun 13;35(9):3398–412. doi: 10.1093/plcell/koad165 (PMC10473229; doi:10.1093/plcell/koad165)
Supplement: koad165_Supplementary_Data [file tpc22.01212.PeerReviewReport.pdf › tpc22.01212.PeerReviewReport.pdf]

Plastid double-strand RNA transgenes trigger phased small RNA-based gene silencing of nuclear-encoded genes

Sébastien Bélanger, Marianne C. Kramer, Hayden A. Payne, Alice Y. Hui, R. Keith, Slotkin, Blake C. Meyers, Jeffrey M. Staub

Corresponding author: Jeffrey M. Staub (jeff@plastomics.com)

Review timeline:

**TPC2022-BR-01212**	Submission received:	December 8, 2022
	1st Decision:	January 31, 2023 *revision requested*
**TPC2022-BR-01212R1**	1st Revision received:	March 21, 2023
	2nd Decision:	May 5, 2023 *accept with minor revision*
**TPC2022-BR-01212R2**	2nd Revision received:	May 15, 2023
	3rd Decision:	May 17, 2023 *acceptance pending, sent to science editor*
	Final acceptance:	June 5, 2023

REPORT: (The report shows the major requests for revision and author responses. Minor comments for revision and miscellaneous correspondence are not included. The original format may not be reflected in this compilation, but the reviewer comments and author responses are not edited, except to correct minor typographical or spelling errors that could be a source of ambiguity.)

TPC2022-BR-01212 1st Editorial decision – *revision requested* January 31, 2023

We have received reviews of your manuscript entitled "Plastid double-strand RNA transgenes trigger small RNA-based gene silencing of nuclear-encoded genes." Thank you for submitting your best work to The Plant Cell. The editorial board agrees that the work you describe is substantive, falls within the scope of the journal, and may become acceptable for publication, pending revision and potential re-review.

We ask you to pay attention to the following points in preparing your revision:

Reviewers 1 and 3 were very enthusiastic about the manuscript and raised largely minor concerns. Reviewer 2 was much more critical about the experimental design, conclusions, and impact. In post-review consultation the three reviewers discussed in more detail some of the experimental concerns. The chief among these were the low number of independent transformants used for analysis. Both reviewers 2 and 3 pointed out that only a single line for PTS39 was analyzed. The reviewer's consensus was that a minimum of two lines is required. PCR or an alternative assay should be used to confirm that integration of the transgenes in the lines analyzed is "clean" at the targeted loci. Another especially important comment to address is the question of whether plastids naturally produce function siRNAs, or is this just confined to trans genes?

The revision should include a detailed response to each point made by each reviewer. Most comments should be addressed by changes to the manuscript and/or new experiments. Convincing rebuttals are also acceptable. We plan to send the revised version to the original three reviewers.

Please note that there may be some reviewer comments (reviewer 2) about novelty or fit for the journal; you may disregard these, as we have determined during pre- and post-review consultations that your work does fit the scope for The Plant Cell.

Please contact us if there are ambiguous comments or if you wish to discuss the revision.

Given the nature of the comments, we are offering you 60 days from when we have issued this decision to complete the revision. If a revision is not returned within this time frame, and if you have not been granted an extension, we will

withdraw the manuscript, which will leave you free to submit the work elsewhere. If you need an extension, we encourage you to contact us at any point before the 60 days have passed.

-- Reviewer comments:

TPC2022-BR-01212R1 1st Revision received March 21, 2023

Editor comments and **author responses**:

Low number of independent transformants used for analysis. Both reviewers 2 and 3 pointed out that only a single line for PTS39 was analyzed. The reviewer's consensus was that a minimum of two lines is required.

We understand the reviewers' consensus opinion that results from two independent chloroplast transformed tobacco lines be reported. The Plastomics company did create two independent transplastomic lines for the PTS39 construct, and in fact, included line PTS39-4 in the Southern blot shown in Supplemental Figure 1. This line also had no obvious phenotype but, unfortunately, seeds from that line were lost during storage prior to our detailed molecular analyses. We can not make additional PTS39 transplastomic lines without significant effort and time, so have now softened and clarified several statements regarding PTS39 data and to address other specific reviewer comments below. We have also modified the legend to Supplementary Figure 1 to indicate the seeds from that line were lost.

We would like to point out that since chloroplast transgene insertion is exclusively via homologous recombination, each independent transplastomic line is identical in its chloroplast genotype, and therefore their phenotypic responses are also uniform - this is quite different from nuclear transgenes that are highly variable in both the insertion site and the phenotype of independent lines, hence the requirement of at least two lines for nuclear transgenes. To illustrate this uniformity, we have sequence confirmed the entire transgenic insert and the junction with flanking non-transformed chloroplast genome sequences in all of the transplastomic lines and now report this in the Methods section on lines 634-637. These results show that each line is a product of perfect homologous recombination, as expected; that is, they are genetically identical.

We have also now also included the sequences of the PDS transgenes as a new Supplemental Table 3 and added a note in the manuscript at lines 628-29. The remainder of the transgenic sequences derive from pJZ199 plasmid from Ralph Bock's lab and is already available in GenBank.

Another especially important comment to address is the question of whether plastids naturally produce <u>functional</u> siRNAs, or is this just confined to trans genes?

To our knowledge, there are no reports of functional plastid siRNAs. Multiple groups have identified small RNAs that map to plastid transcribed regions that have been shown to generally correlate with binding sites for plastid PPR or TPR RNA protein binding sites. While some of these RNA footprints appear to be conserved across several plant species, there is no evidence that these small RNAs have a distinct function and are thought currently to be a consequence of being protected from degradation by those RNA binding proteins. As mentioned below, we have now included a brief statement and cite a relevant review in the Results section (lines 292-295). Consistent with this, our analysis of small RNAs in partially purified plastid fractions likewise did not find any enrichment for known 21-24 nucleotide sRNA classes or phased siRNAs, as already discussed in the Results section.

Reviewer comments and **author responses**:

<u>Reviewer #1:</u>

This is an excellent manuscript that changes the way we think about chloroplasts. Until now chloroplasts are viewed as a conservative organelle, with a selective protein import machinery, but no export of proteins or RNA. This manuscript reveals that there is at least a backdoor with a massive export of RNA, possibly at the time of organelle degradation, that feeds into the small RNA processing machinery. PTS40 plants expressing dsRNA in the chloroplast are the source of 21-nt siRNAs, reducing the levels of PDS mRNA via degradation. Plants expressing the single-stranded sense strand (PTS38) accumulate exclusively sense strand fragments of a broad size range, and possibly act through inhibition of translation. Findings reported here pave the way to chloroplast (plastid) control of nuclear gene expression, a conceptually new regulatory pathway.

The experiments are high quality, fully supporting the claim of chloroplasts being the source of regulatory RNAs. I suggest only minor changes:

(1) Line 328, Table 1. For the benefit of the general reader, define/explain what is "phasing score", and what is its significance here.

We have now added a definition of Phasing Score to the Methods section at lines 766-68.

(2) Title: delete "double-stranded" because both sense strand ssRNA and dsRNA silence nuclear encoded genes, although by a different mechanism

The majority of the manuscript focuses on phasiRNAs produced from double-stranded RNA constructs. Since we do not fully understand the mechanism of silencing by the sense-strand construct, per reviewers comments (see below), we have softened some statements in that regard. Therefore, we suggest keeping "double-stranded" and have added "phased" small RNAs to the title to emphasize our main finding.

(3) Line 263. Title incomplete

Thank you for pointing this out, we have added the missing word "RNA" to the section heading.

(4) Line 158 cites Supplementary Figure 1. Section D with T0 in the greenhouse. Section D of Figure is missing.

Thank you for pointing this out. Reference to Supplemental Figure 1D has been removed.

Reviewer #2:

In this ms, the authors report that expressing dsRNA in the plastids results in the (moderate) accumulation of siRNA in the cytoplasm, which can trigger down-regulation of homologous endogenous targets, similar to siRNAs produced from nuclear-encoded dsRNA. This is an interesting finding, but the results are very preliminary (see comments 4, 5, 6, 8, 9). Also, given the complexity to transform plastids, the advantage of producing siRNA from plastid-encoded dsRNA is not clear. Moreover, the fact that RNA exiting the plastids have regulatory roles has already been shown.

Major comments

(1) In the introduction the authors say that "high-level accumulation and efficacy of long insecticidal dsRNA in plastids was attributed to the lack of dsRNA processing in plastids due to the absence of the RNAi machinery". They also say that "plastids import up to ~3000 nuclear-encoded proteins". At least, they should say if DCLs are, or are not, among the proteins that enter in the plastids?

We have already cited numerous publications in the Introduction and Discussion of the manuscript that all claim lack of the RNAi machinery and gene silencing components in plastids, including DCLs (see lines 42-44, 394-396), in plastids. Consistent with those reports, we do not see evidence of distinct small RNA classes in partially purified plastid fractions from wild-type plants. Inspection of the subcellular proteome within the Plant Proteome Database (http://ppdb.tc.cornell.edu/) did not identify any DCL proteins with homology to known DCLs involved in gene silencing (note a plastid-localized protein, named DCL (DUF3223), encodes a protein Defective Chloroplasts and Plant Leaves).

(2) In the introduction the authors also say that "intact plastid-encoded RNAs have not been observed outside of the organelle, though plastid-encoded tRNA fragments have been observed in the cytoplasm and suggested to be involved in regulatory processes. These observations suggest a means by which plastid DNA or RNA can exit the organelle to function in other cellular compartments". If the regulatory function of RNAs exiting from plastids is already known, the author's finding that dsRNA exiting plastids to produce functional siRNA is hard to consider as a breakthrough.

As customary, we cite the available literature that suggests plastid-derived nucleic acids may be found in other cellular compartments. Since there is no clear role for the plastid-derived tRNA fragments that have been found in the cytoplasm, we have deleted the part of the sentence (previously on line 68) that stated they are "suggested to be involved in regulatory processes". Since the production of phasiRNAs from a plastid construct was completely unexpected and shown to be involved in silencing a nuclear-encoded gene, we believe this result may be

considered a breakthrough.

(3) In the results section, the authors say that "highly efficient dsRNA-mediated knockdown of PDS was confirmed by qRT-PCR and small RNA sequencing, which indicated processing of the plastid-expressed dsRNA into 21-nt phasiRNA". This is a wrong usage of the phasiRNA terminology. PhasiRNA are secondary siRNAs produced by genes primarily targeted by a 22-nt miRNA or siRNA. Here, the siRNAs that are produced derive exclusively from the inducing PDS dsRNA, not from the PDS targeted by these siRNAs. Therefore, they are regular siRNAs, not phasiRNAs.

We appreciate the reviewer's viewpoint, but we would argue that the most relevant attribute of a set of "phasiRNAs" is that they are phased, which is the case in our data. A recent example of phasiRNAs which are not secondary but are phased is on the cover of a recent issue of the journal Science in which an inverted repeat in Mimulus species generates phasiRNAs but there is also no small RNA trigger (see doi: 10.1126/science.adf1323) and in our previous report in diverse monocots (see doi: 10.1101/gr.228163.117). We have now referred to these reports in the Discussion section at lines 335-37.

(4) The authors claim that expressing an antisense PDS RNA has no effect, whereas sense PDS RNA and PDS dsRNA cause PDS silencing. However, the number of lines analyzed is way below the number required for a publication. Based on Figures 1, 2 and Sup F1C, it seems that only one antisense line, two sense lines and two dsRNA lines were analyzed. At least five of each is mandatory.

We address the number of lines above and emphasize that since plastid integration occurs by homologous recombination, independent events are genetically identical and as a result, phenotypically the same. The number of transgenic lines typically assessed is a requirement for nuclear transformation, for which no two independent transgenic events are the same. We have also clarified in the Discussion that the very low levels of small RNAs and a weak phasiRNA score does accumulate from the antisense RNA construct (lines 347-349), as shown in Table 1.

(5) The authors claim that "highly efficient dsRNA-mediated knockdown of PDS was confirmed by qRT-PCR". However, Fig2A and 2B show that the decrease in PDS mRNA accumulation is less than two-fold. This is not "highly efficient". Moreover, how can this mild decrease cause bleaching considering that plants heterozygous for a pds mutation have no bleaching phenotype ?

We agree with the reviewers assertion that the less than 2-fold decrease in PDS mRNA is not "highly efficient" and we have removed that statement. We also agree that it is unclear why the PDS bleaching phenotype is so severe in our dsRNA lines, but we interpret this as a demonstration of the efficacy of the phasiRNAs reducing the levels of the PDS transcripts below a threshold, perhaps with greater efficacy in specific cell layers.

(6) In Fig2C the northern blot hybridized with an antisense probe should detect the endogenous PDS mRNA in WT controls, which is not the case. How do the authors explain this ?

We had already stated in the legend to Figure 3 that the nuclear encoded PDS genes are expressed at much lower levels than the plastid transgenes and so are not seen on the blot at that level of exposure. We have moved that statement from the figure legend to the Results section to make this more clear. Previous publications have noted that dsRNAs expressed in plastids can accumulate very high levels of RNA, up to 0.8% of total cellular RNA. Our edits are on lines 243-46.

(7) The authors say that "reduction of nuclear-encoded PDS mRNA levels via plastid transgenic RNAs suggests that the latter can enter the PTGS pathway, which is characterized by the presence of 21 to 24 nt siRNAs". This is incorrect : only 21 and 22 nt siRNA cause PTGS.

We have corrected the statement on lines 249-250 to, "characterized by the presence of 21 or 22 nt siRNAs".

(8) The level of PDS siRNA is very low (200 rpm), compared with the level of siRNAs accumulating in plants expressing <u>some other random?</u> nuclear-encoded dsRNA. This suggests that the export of dsRNA from plastids is very inefficient or that dsRNA that exit the plastids are altered. PDS is a very sensitive target so I wonder if plastid-engineered RNAi would work against other targets.

We agree with the statement that PDS siRNAs are of relatively low abundance. Some of the plastid dsRNA transgenes with no cognate target in plants accumulate siRNAs to a much higher level (up to 1300 rpm) while others

also accumulate only low levels. We have now raised this issue briefly in the Discussion in relation to the PTS39 line (lines 349-351) and in relation to the developmental stage of the plastid (lines 366-69).

(9) The conclusions are generally over-estimated. For example, the effect of sense RNA is puzzling, and the authors don't really understand what happens in these plants. However, they conclude: "we also observed apparent translational inhibition from sense-strand RNAs in the PTS38 line, as evidenced by pigment deficient plants with no PDS RNA knockdown via qRT-PCR". There is no evidence for translational inhibition. Another example : on line 309, the authors write "is a universal phenomenon" after testing only 2 exogenous dsRNA. "likely is a general phenomenon" would be greatly sufficient. Finally, the authors say that "plant nuclear-encoded dsRNA transgenes may lose efficacy due to processing of their dsRNA in the cytoplasm to 21 - 24 nt small interfering RNAs (siRNAs), which are not efficiently taken up by insect gut cells". To test this hypothesis and determine if the fraction of dsRNA that are processed by the DCLs limits their efficacy against insects, it is necessary to compare the effect of anti-insect dsRNA in wt plants versus dcl mutants

We agree that the effect of sense RNA is not understood and have softened our statement. Likewise, the lack of bleaching from the PTS39 antisense line is noteworthy. We have now briefly discussed both of these aspects (lines 345-51).

We have also softened our statement at line 296 per the reviewers guidance to a "general phenomenon", and in the Abstract line 16.

Regarding the reviewers last point of whether the fraction of dsRNA that are processed by DCLs limits their efficacy against insects, we believe this is beyond the scope of our report and addressed by the citations we have already included in the Introduction of the manuscript.

<u>Minor comments</u>

(1) Sup F1D is missing

Done. Reference removed.

(2) Figures don't come in order in the text (1A, 2D, 1D, 1B, 1C, 2A, 2B, 2C)

Done. Figures are now described in order. We have also created a new Supplementary Figure 2 to more clearly show the confocal imaging results.

<u>Reviewer #3:</u>

In this paper Belanger et al a novel, very interesting mechanism for the use of chloroplast as a source for dsRNA as a substrate for cytoplasmic PTGS is described. The data showing that dsRNA derived from transgenic chloroplasts are found in cytoplasm and subjected to cytoplasmic PTGS machinery are clear. Moreover, chloroplast derived dsRNA/stem loop RNA do not require the presence of endogenous nuclear cognate gene to produce siRNAs and phasiRNAs.
The work is important, novel however, for mechanistic details still intriguing and leaving many opened questions. Below I raised my major and minor concerns.

Major concerns:

(1) In your or others NGS data can you detect any evidence that the mechanism you describe is functionally/biologically present and important? If not - your work provide fantastic tools for plant gene expression manipulation however, it would be much more spectacular if such a mechanism is used by plants in some physiological conditions.

As we state above, our data and the previously published reports show no evidence for phased siRNAs from endogenous plastid transcripts, though we can not rule out such a mechanism under other as yet untested physiological conditions.

The only other well characterized small RNAs in plastids are RNA-binding footprints that are protected from degradation. We have also now included in the Results section (line 292-295) that we made no attempt to identify previously observed footprint small RNAs with no known function.

(2) It is mysterious why in the PTS39 lines there is no silencing. I would expect that if translational inhibition is more likely to occur In PDS silencing in the PTS38 lines - antisense should work even better by making dsRNA regions in mRNA during translation?

We agree this is an interesting point and have now added brief comments in the Discussion starting at line 345-51, as also discussed above.

Minor concerns

(1) Supplemental Fig,1D is missing

Thank you for pointing this out. Reference to Supplemental Figure 1D has been removed.

(2) Mesophyll cells of PTS39 with large chloroplasts should be shown in supplementary material

We have now included confocal images of both mesophyll and epidermal cells from the PTS39 line. We have also moved the figure to new Supplemental Figure 2 so that we can enlarge the images and show more detail.

(3) What is the difference between epidermal and mesophyll cells that PDS knockdown does not occur there?

It is unclear if PDS knockdown is not occurring in epidermal cells - we only know that the morphology of the plastids in epidermal cells appears unchanged from the wild-type pattern. We speculate that epidermal cells may be relatively unaffected because this single-cell layer is known to have very little accumulation of chlorophyll or other pigments, to enable light to penetrate.

(4) Fig. 2E is not of good quality and difficult to interpret. Can you use better resolution? Why is there a dense packing of chloroplasts in PTS40 plants epidermis? This is not commented on in the text. Chloroplast pairs arrested in their division in the mutant lines are difficult to see and in WT plants it is also difficult to see that there are no such pairs, if I am correctly analysing the data.

We have created new Supplemental Figure 2 with larger panels and displayed the images in black and white to enhance their resolution. The dense packing of cells seen especially in PTS38 and PTS40 mesophyll images are due to Z-stack image processing that increased the depth of view to slightly greater than a single cell layer. The leaf cells in pigment deficient leaves of PTS38 and PTS40 lines are slightly disordered, also contributing to the perception of dense packing in these 3D processed images. The paired plastids are also now more easily visible, especially in the PTS40 line. We have now included this information in the Supplemental Figure legend.

(5) Fig.3 Why only one PTS39 line is shown? There should be a second line presented as well. Text describing the level of PDS1 and PDS2 is not clearly written and does not fit to Fig.3 A and Fig.3B panels. I agree that in 12 and 24-day-old seedlings PDS1 and PDS2 mRNAs are reduced by half in PTS40 lines. I disagree with the statement, that the level of PDS1 mRNA is slightly reduced in PTS38 lines. It is not. In 24 day-old seedling the result for PTS40 lines is clear but the results for two PTS38 lines are completely the reverse concerning PDS1 and PDS2 mRNA levels. The whole paragraph should be rewritten.

We have discussed the lack of availability of a second PTS39 line above.

We agree that the text describing PDS1 and PDS2 in the qRT-PCR section was not clearly written and we have reworked that section of the manuscript, at lines 203-222.

(6) Fig.3C - northern blot of chloroplast PDS1 sense/antisense transcripts: I can accept the interpretation of read through transcripts but this will mean that bacterial terminator in PTS38 mutants is not operating efficiently in your chloroplast constructs. Why does it operate efficiently in PTS39 lines? This should be commented. What are red arrows and star in the 3C figure?

The red arrows represent readthrough transcripts and the red asterisk represents the PDS-only transcript. We have now added this note to the figure legend.

In the case of PTS38 sense lines, based on the size of the readthrough transcripts, these are likely being stabilized by elements in the neighboring aadA transgene (for example the 3'-UTR stem/loop) and thus accumulate to a high level. In contrast, the transcripts in the PTS39 line read through to the neighboring plastid genome, where it is possible that these are likely less stable due to the absence of or distance from a suitable stabilizing element. We

have noted this in the Results section starting at line 241-43.

(7) Line 263 The title is missing RNA word (Processing of plastid transgene-encoded small in the cytoplasm) should be small RNA

Done. We have added the missing word "RNA" to the section heading.

(8) If in the case of PTS38 line translational inhibition is suggested as possible mechanism for bleaching phenotype (no evidence to downregulate PDS1/2 mRNA levels at least in 12 day-old mutant lines) It would be good to see the western analysis of PDS1/2 proteins downregulation in at least PTS38 lines.

We agree that it would be interesting to better understand the bleaching mechanism in the PTS38 line. However, comparative protein analysis across our pigment deficient lines or with a green plant would be very difficult to normalize and interpret. Comparison of the PTS38 line to a wild-type line is not feasible because of the dramatic differences in total protein between a green plant and the pigment deficient lines. Comparison between PTS38 and PTS40 bleached lines could be performed, however, because a reduction of PDS is expected in the PTS40 line, any result in the PTS38 line would be ambiguous and difficult to interpret. Since we believe that the mechanism of bleaching in the PTS38 line is beyond the scope of this manuscript, we respectfully believe a western blot would not significantly enhance our understanding of that line in case.

TPC2022-BR-01212R1	2nd Editorial decision – *accept with minor revision*	May 5, 2023

We have received reviews of your manuscript entitled "Plastid double-strand RNA transgenes trigger phased small RNA-based gene silencing of nuclear-encoded genes." On the basis of the advice received, the board of reviewing editors would like to accept your manuscript for publication in The Plant Cell. This acceptance is contingent on revision based on the comments of our reviewers. In particular, please consider the following:

TPC2022-BR-01212R2	2nd Revision received	May 15, 2023

We thank Reviewers 1 and 3 for their continued support of our manuscript. We appreciate the concerns from Reviewer 2 and have tried to further address those concerns via additional edits to the manuscript. As a result, we have now added a new Supplementary Figure and expanded some of the discussion in the Results and Discussion sections of the text. We hope the manuscript is now ready for publication in The Plant Cell.

Reviewer comments and **author responses**:

Reviewer #1:

This is a revised manuscript in which the reviewer's criticisms have been satisfactorily addressed.

Reviewer #2:

This revised manuscript shows very little improvement compared to the first submission. Not only it does not contain any new data, but it does not address my comments in a satisfying way. In my opinion, the results are interesting but preliminary. The manuscript is short and largely over-interpreted. Additional experiments/data are needed for publication.

While we appreciate the Reviewer's overall concerns that the results are preliminary, we believe our novel and first report of plastid-derived phased siRNAs involved in nuclear gene silencing warrants publication as a Breakthrough Report. Based on the guidelines to authors of such reports, "these papers are not required to be a 'full story' but they should be especially novel and exciting and open a new area of investigation in the field". We believe that our work meets these criteria.

1) siRNA nomenclature
In the introduction the authors say: "siRNA processing occurs on membrane-bound polysomes to trigger the production of secondary siRNAs that are generated on the rough endoplasmic reticulum (Li et al., 2016). These secondary siRNAs have a characteristic "phased" pattern, resulting from successive cleavage by a DICER-LIKE

protein on the double-stranded RNA substrate. In many plants, DICER-LIKE4 (DCL4) generates 21-nt phased siRNAs (phasiRNAs), which are a characteristic outcome of PTGS (Liu et al., 2020)."
The last and corresponding author is not a small RNA person, but two of the authors are expert PIs in the field so I am very surprised that they are fine with the way the paper introduces siRNAs. The term phasi-RNAs is restricted to siRNAs that are produced from RNA precursors that are first targeted by a small RNA (miRNA or siRNA). This is not the way siRNAs are produced from dsRNA precursors that are directly produced as dsRNA molecules without being targeted by a small RNA (from an inverted-repeat or by convergent transcription). The fact that siRNAs are phased simply reflects the processive activity of DCLs. The authors should stick to a nomenclature that has been established for years and not introduce confusion in the readers' mind by calling the siRNAs they detect phasi-RNAs without knowing their mode of biogenesis. If they want, they could coin them plastid-derived siRNAs.

We appreciate the Reviewer's comments and we have already briefly discussed the recently published examples of phasiRNAs generated from dsRNA, apparently without AGO-directed cleavage (on lines 334-337 of the Discussion). To further clarify the observation for our plastid-derived phasiRNAs, we have now referred to these as "untriggered phasiRNAs" in the same section of the Discussion (lines 337-340). We agree that further research is needed to fully understand their biogenesis.

2) Number of independent transformants
In their rebuttal letter, the authors say: "since chloroplast transgene insertion is exclusively via homologous recombination, each independent transplastomic line is identical in its
chloroplast genotype, and therefore their phenotypic responses are also uniform".
This is theorical. Figure 3B shows clearly that the two PTS38 transformants behave differently, one being above WT, the other below.

The Reviewer's comments are noted. We had already noted the variability seen in lines from PTS38 in the Results section. Furthermore, the manuscript already discusses at length that the mechanism of PDS gene silencing in the PTS38 line is unclear, while the maternally-inherited bleaching phenotype is very clear (for example, Results section lines 209-215 and the Discussion, lines 341-347. Figure 3B clearly shows that the multiple PTS40 lines that make phasiRNAs have uniformly decreased PDS mRNA levels at all timepoints, which is the emphasis of Figure 3B and the rest of the manuscript.

3) Silencing mechanism
The authors report that: "In 12-day-old seedlings, when cotyledons of PTS38 and PTS40 lines are uniformly bleached (Figure 1C), the level of PDS1 and PDS2 mRNA are significantly reduced in the PTS40 dsRNA lines to ~50% of wild-type levels, while no change in PDS mRNA was observed in the PTS38 lines (Figure 3A). In 24-day-old seedlings of the PTS40 line (Figure 3B), the mRNA levels of the nuclear-encoded PDS1 and PDS2 remain reduced compared to wild-type, indicating that reversion of the bleaching phenotype is independent of PDS mRNA levels. In contrast, the PDS mRNA levels in the PTS38 lines appear more variable at this later developmental stage (Figure 3B)."
The only conclusion of this analysis is that there is no correlation between the bleaching phenotype and the PDS mRNA level, even in lines PTS40. The maximum reduction in PDS mRNA level is two-fold, which cannot explain the bleaching phenotype (in Arabidopsis, a plant heterozygote for a pds null mutation has two times less PDS but looks WT). Clearly, there is something beyond this two-fold reduction of PDS mRNA level. This may be related to translational inhibition, but it is not investigated at all. Additional experiments are crucially needed here.

We are unsure if the Reviewer's extrapolation of Arabidopsis mutant phenotypes to tobacco plastid transformants is a valid comparison. However, we note the Reviewer's point that translational inhibition may be involved. To account for this, we have added clarification to the manuscript in the Results section at lines 221-222 that "additional levels of regulation including translation control" of PDS may be involved.

4) Silencing efficiency
The authors acknowledge that "In 24-day-old seedlings of the PTS40 line (Figure 3B), the mRNA levels of the nuclear-encoded PDS1 and PDS2 remain reduced compared to wild-type, indicating that reversion of the bleaching phenotype is independent of PDS mRNA levels."

The silencing effect caused by plastid-expressed dsRNA appears unstable. Based on the only target analyzed (PDS), this method cannot be considered as a reliable method for silencing nuclear genes. The authors could at least test if the siRNAs expressed from dsRNA that are not homologous to the nuclear genome can silence exogenously introduced homologous targets.

Our manuscript highlights the production of phasiRNAs from 5 different plastid dsRNA constructs representing a nuclear encoded gene (PDS, a homologous target) and 4 nonhomologous insect target genes. Thus, we conclude that phasiRNA production in the cytoplasm is an expectation from plastid-expressed dsRNA genes, and is the major conclusion of our manuscript. As is well known in the art, the success and extent of silencing of new gene targets can differ depending on the introduced target sequences and target genes, and would require undue experimentation for a Breakthrough Report. We note, however, that we introduced a second PDS seed sequence expressed as a dsRNA in plastids that was successful in PDS gene silencing (PTS41, PDS seed sequences 931-1210 based on the cDNA sequence). Silencing of PDS in this line occurred but was relatively weak compared to PTS40 and was not used in our small RNA analyses due to limitations in resources and funding, and so was not included in the manuscript.

5) Biological significance

Based on the authors data and on previous publications, it is clear that plastid RNAs can exit the organelle. The question is what happens to these RNAs and if there is a biological significance to this exit in wild-type plants. Indeed, it is an artificial situation created by the authors that suggests that RNAs exiting plastids can interfere with the expression of nuclear genes. Whether nuclear genes are regulated by small RNAs derived from wild-type plastids is the key question. The authors should determine if there are siRNAs that do not match with the nuclear genome but match with the plastid genome. If so, they should mine PAREseq data to determine if these plastid-derived siRNAs have a regulatory role and cleave nuclear-encoded mRNAs. If it is not the case, and if plastids produce siRNAs only when engineered with dsRNA-producing transgenes, the paper loses its interest, except if the authors explain what is the advantage of such a strategy compared with classical nuclear RNAi.

The Reviewer raises interesting comments and suggestions for new experiments, some of which may be investigated in the future. However, we disagree with the assertion that our results would be less interesting if endogenous genes are not regulated by plastid small RNAs. The advantage of our strategy is already outlined as a new approach to silence nuclear genes from maternally-inherited plastids, with all of the advantages of plastid transformation discussed in the Introduction and Discussion sections of the manuscript.

Minor comments

I previously mentioned that the northern blot hybridized with an antisense probe (Figure 3C) should detect the endogenous PDS mRNA in WT controls. The authors answered that the plastid PDS RNAs are more abundant than the nuclear PDS mRNA. Fine, but they could at least show a longer exposure of the hybridization in the supplemental material.

The filter used in the northern blot in Figure 3 was over-exposed at the time of the experiment in an attempt to visualize the nuclear-encoded PDS mRNA. Due to the vast abundance of the plastid-expressed RNAs and bleed-over of the hybridization signal into adjacent lanes, any PDS signal in the wild type lanes was obscured. Based on reported accumulation of plastid-expressed dsRNAs to as high as 1% total cellular RNA, with mRNAs accumulating to typically at least 100-fold less than that, the result was not unexpected. However, we have now included in the manuscript a new Supplementary Figure 3 to show the darkest exposures of the northern blot that were taken at the time of the experiments. The numbering of the subsequent Supplementary Figures has been adjusted accordingly.

<u>Reviewer #3:</u>

This is a second round of revision of this MS for me and I carefully read Author's responses and additions to the main text and supplementary material. Although I raised concerns for the lack of the second transgenic PTS39 line I am satisfied with the Author's explanation and this is now fine with me. The second concern dealing with the the western analysis of the PDS protein is also satisfactory. All other remarks have been addressed and missing information/explanation is added. I have no other questions, I regard the MS as important, original and novel.

We are pleased to inform you that your paper entitled "Plastid double-strand RNA transgenes trigger phased small RNA-based gene silencing of nuclear-encoded genes" has been accepted for publication in The Plant Cell, pending a final editorial review by a science editor. At this stage, your manuscript will be evaluated by a science editor with respect to its presentation of scientific content, compliance with journal policies, and presentation for a broad readership.

Final acceptance from Science Editor **June 5, 2023**
